# Exploring *Monacha cantiana* (Montagu, 1803) phylogeography: cryptic lineages and new insights into the origin of the English populations (Eupulmonata, Stylommatophora, Hygromiidae)

**DOI:** 10.3897/zookeys.765.24386

**Published:** 2018-06-06

**Authors:** Joanna R. Pieńkowska, Giuseppe Manganelli, Folco Giusti, Alessandro Hallgass, Andrzej Lesicki

**Affiliations:** 1 Department of Cell Biology, Institute of Experimental Biology, Faculty of Biology, Adam Mickiewicz University in Poznan; Umultowska 89, 61-614 Poznań, Poland; 2 Dipartimento di Scienze Fisiche, della Terra e dell’Ambiente, Universitá di Siena, Via Mattioli 4, 53100 Siena, Italy

**Keywords:** 16SrDNA, COI, H3, ITS2, molecular features, reproductive system, Roman origin, shell, structure, species distribution

## Abstract

Molecular analysis of nucleotide sequences of mitochondrial cytochrome oxidase subunit 1 (COI) and 16S ribosomal DNA (16SrDNA) as well as nuclear histone 3 (H3) and internal transcribed spacer 2 of rDNA (ITS2) gene fragments together with morphological analysis of shell and genitalia features showed that English, French and Italian populations usually assigned to *Monacha cantiana* consist of four distinct lineages (CAN-1, CAN-2, CAN-3, CAN-4). One of these lineages (CAN-1) included most of the UK (five sites) and Italian (five sites) populations examined. Three other lineages represented populations from two sites in northern Italy (CAN-2), three sites in northern Italy and Austria (CAN-3), and two sites in south-eastern France (CAN-4). The taxonomic and nomenclatural setting is only currently available for lineages CAN-1 and CAN-4; a definitive frame for the other two requires much more research. The lineage CAN-1 corresponds to the true *M. cantiana* (Montagu, 1803) because it is the only one that includes topotypical English populations. The relationships and genetic distances support the hypothesis of the Italian origin of this lineage which was probably introduced to England by the Romans. The lineage CAN-4 is attributed to *M. cemenelea* (Risso, 1826), for which a neotype has been designated and deposited. Its diagnostic sequences of COI, 16SrDNA, H3 and ITS2 genes have also been deposited in GenBank. Molecular and morphological (shell and genitalia) features showed that *M. parumcincta* (Rossmässler, 1834) is a distinct taxon from the *M. cantiana* lineages.

## Introduction

*Monacha* is a diverse genus of the trochuline hygromiids widespread in the western Palaearctic from western Europe to north Africa, Iran, and Arabia. It includes a large number of nominal species and shows its highest diversity in the eastern sector of southern Europe and in Turkey (Hausdorf 2000a, 2000b, Welter-Schultes 2012, Neiber and Hausdorf 2017).

*Monacha cantiana* (Montagu, 1803) is one of the westernmost species. It is a medium-sized land snail living among grass in open habitats such as grasslands, pastures, cultivated and uncultivated fields or forest edges and clearings. Its geographical distribution, probably southern European in origin, was partly shaped by anthropochorous dispersal which helped the species to reach north-western Europe. For example, in the British Isles it is considered to have been introduced and this hypothesis is supported by the absence of a Holocene fossil record in England older than the third century AD (Kerney et al. 1964, Kerney 1970, 1999, Evans 1972).

The aim of the present research was: (1) to study molecular and morphological (shell and genitalia) variation of the species in order to explore its phylogeography and detect any geographical patterns; (2) to investigate relationships between molecular and morphological variability in order to characterise clades recovered by molecular study; (3) to test the hypothesis that the English populations originated from introduced propagules.

## Material and methods

### Taxonomic sample

Our analysis considered a number of populations of *Monacha cantiana*, mainly from Italy and England, that represent its gross morphological, geographical, and ecological variability. Some sequences deposited in GenBank were also considered for the molecular analysis. One population from the type locality of *Theba cemenelea* Risso, 1826 a taxon regarded as a junior synonym, subspecies or species, slightly distinct from *M. cantiana*, was also included. For comparison, two other *Monacha* species were used in the molecular analysis: *Monacha cartusiana* (Müller, 1774) and *M. parumcincta* (Rossmässler, 1834). The latter was also used in the morphological analysis. While *M. cartusiana* is a well-established taxon, the taxonomic and nomenclatural status of *M. parumcincta* is still disputed, e.g. conspecificity of Italian and Balkan populations, authorship to Rossmässler, 1834 or Menke, 1828 (see Forcart 1965, Manganelli et al. 1995, Welter-Schultes 2012).

### Material examined

Material examined is listed as follows, when possible: geographic coordinates of locality, locality (country, region, site, municipality and province), collector(s), date, number of specimens and collection in which material is kept in parenthesis (Table 1). Collection acronyms: FGC (F. Giusti collection, Dipartimento di Scienze Fisiche, della Terra e dell’Ambiente, Università di Siena, Italy); DCBC (Department of Cell Biology Collection, Adam Mickiewicz University, Poznań, Poland).

**Table 1.** List of localities of the specimens of *Monacha cantiana* (CAN-1 to CAN-4), *M. parumcincta* and *M. cartusiana* used for molecular and morphological (SH shell, AN genitalia) research.

Localities				Clade	Revised taxonomy	COI			16SrDNA			H3			ITS2			PCA and RDA	Figs
No.	coordinates	country and site	collector / date / no. of specimens (collection)			new haplotype	no. sps	GenBank ##	new haplotype	no. sps	GenBank ##	new common sequence	no. sps	GenBank ##	new common sequence	no. sps	GenBank ##		
1.	53°31'29"N, 01°27'54"W	United Kingdom, Barrow near Barnsley	R.A.D. Cameron / 10.2011 / 5 (FGC 40329) (Pieńkowska et al. 2015)	CAN-1	*M. cantiana*			KM247375			KM247390							SH, AN	20, 22–23, 25
						UK-COI 1	4	MG208884	UK-16S 1	5	MG208966	UK-H3 1	3	MG209031	UK-ITS2 2	3	MH137963		
								MG208885			MG208967			MG209032			MH137964		
								MG208886			MG208968			MG209033			MH137965		
								MG208887			MG208969								
											MG208970								
2.	51°30'30"N, 00°15'38"W	United Kingdom, East Acton near London	M. Proćków / 07.06.2010 / 3 (DCBC & FGC 42965)	CAN-1	*M. cantiana*	UK-COI 1	3	MG208888	UK-16S 1	3	MG208961							SH	21, 24
								MG208889			MG208962								
								MG208890			MG208963								
3.	Not available	United Kingdom, Cambridge (old material)	F. Giusti / 1981 / 3 (FGC 23773)	CAN-1	*M. cantiana*	UK-COI 2	1	MG208891	UK-16S 1	1	MG208972	UK-H3 2	1	MG209034				SH, AN	
						UK-COI 3	1	MG208892	UK-16S 1	2	MG208973								
											MG208974								
4.	53°25'04.2"N, 01°24'00.5"W	United Kingdom, Rotherham	R.A.D. Cameron / 07.2015 / 7 (DCBC)	CAN-1	*M. cantiana*	UK-COI 1	1	MG208883							UK-ITS2 2	1	MH137966		
						UK-COI 4	1	MG208893	UK-16S 1	2	MG208960	UK-H3 3	1	MG209035	UK-ITS2 1	1	MH137967		
											MG208964								
						UK-COI 5	1	MG208894											
						UK-COI 6	1	MG208895											
						UK-COI 7	2	MG208897							UK-ITS2 2	2	MH137968		
								MG208898	UK-16S 2	1	MG208975	UK-H3 4	1	MG209037			MH137969		
						UK-COI 8	1	MG208900											
5.	53°24'49.1"N, 01°24'36.6"W	United Kingdom, Sheffield	R.A.D. Cameron / 07.2015 / 6 (DCBC)	CAN-1	*M. cantiana*	UK-COI 6	1	MG208896							UK-ITS2 2	1	MH137970		
						UK-COI 7	1	MG208899	UK-16S 2	1	MG208976	UK-H3 5	1	MG209038	UK-ITS2 2	1	MH137971		
						UK-COI 9	1	MG208901	UK-16S 1	1	MG208965								
						UK-COI 10	1	MG208902											
5.	53°24'49.1"N, 01°24'36.6"W	United Kingdom, Sheffield	R.A.D. Cameron / 07.2015 / 6 (DCBC)	CAN-1	*M. cantiana*	UK-COI 11	1	MG208903											
						UK-COI 12	1	MG208904	UK-16S 1	1	MG208971	UK-H3 3	1	MG209036					
6.	42°28'41.05"N, 13°05'09.46"E	Italy, Latium, Gole del Velino, near Sigillo (Posta, Rieti)	A. Hallgass / 30.09.2012 / 8 (FGC 42960)	CAN-1	*M. cantiana*	IT-COI 1	4	MG208905	IT-16S 1	4	MG208977	IT-H3 1	1	MG209039	IT-ITS2 4	1	MH137972	SH, AN	9, 26, 28–30
								MG208906			MG208979	IT-H3 5	1	MG209041					
								MG208907			MG208980								
								MG208908			MG208981								
						IT-COI 2	3	MG208910	IT-16S 1	4	MG208978	IT-H3 3	1	MG209042					
								MG208911			MG208982								
								MG208912			MG208983								
											MG208984								
7.	Not available	Italy, Tuscany, Elba Island, Sant’Ilario in Campo (Livorno)	F. Giusti / 19.02.1974 / 1 (FGC 23586)	CAN-1	*M. cantiana*	IT-COI 2	1	MG208913										SH, AN	10
8.	42°02'51.18"N, 12°54'19.64"E	Italy, Latium, Valle dell’Aniene (Roccagiovine, Rome)	A. Hallgass / 20.10.2013 / 6 (FGC 42973)	CAN-1	*M. cantiana*	IT-COI 3	3	MG208915	IT-16S 1	3	MG208985	IT-H3 6	1	MG209045	IT-ITS2 2	1	MH137973	SH, AN	8
								MG208916			MG208987	IT-H3 7	1	MG209046	IT-ITS2 3	2	MH137974		
								MG208917			MG208989	IT-H3 8	1	MG209047			MH137975		
						IT-COI 4	1	MG208918	IT-16S 1	1	MG208986								
						IT-COI 5	1	MG208919	IT-16S 1	1	MG208988								
						IT-COI 6	1	MG208920	IT-16S 2	1	MG208995								
9.	42°43'39.87"N, 13°16'01.44"E	Italy, Latium, Valle del Tronto (Accumoli, Rieti)	A. Hallgass / 30.09.2012 / 4 (FGC 42963)	CAN-1	*M. cantiana*	IT-COI 1	1	MG208909	IT-16S 1	1	MG208992							SH	
						IT-COI 2	1	MG208914	IT-16S 1	1	MG208991								
						IT-COI 7	2	MG208921	IT-16S 1	2	MG208990	IT-H3 3	2	MG209043	IT-ITS2 1	2	MH137976		
								MG208922			MG208993			MG209044			MH137977		
10.	42°07'53.39"N, 13°01'39.81"E	Italy, Latium, Valle del Turano, near Turania (Rieti)	A. Hallgass / 04.11.2013 / 2 (FGC 42969)	CAN-1	*M. cantiana*	IT-COI 7	1	MG208923	IT-16S 1	1	MG208994	IT-H3 4	1	MG209048	IT-ITS2 5	1	MH137978	SH, AN	11, 27
						IT-COI 8	1	MG208924											
11.	43°22'59.9"N, 02°59'00.0"W	Spain, Sopelana, Pais Vasco	unknown / (SP164) (Razkin et al. 2015; Neiber and Hausdorf 2015)	CAN-1	*M. cantiana*			KX507234			KJ458539								
											KX495428								
12.	45°11'59.85"N, 10°58'49.30"E	Italy, Venetum, Sorgà (Verona)	A. Hallgass / 09.2012 / 6 (FGC 42964)	CAN-2	*M. cantiana*	IT-COI 9	3	MG208925	IT-16S 3	2	MG208996	IT-H3 9	1	MG209050	IT-ITS2 7	1	MH137979	SH, AN	12, 36–39
								MG208926			MG208997								
								MG208927											
						IT-COI 10	3	MG208928	IT-16S 4	4	MG208998				IT-ITS2 6	1	MH137980		
								MG208929			MG208999								
								MG208930			MG209000								
											MG209001	IT-H3 4	1	MG209049					
13.	45°31'28.95"N, 10°21'35.75"E	Italy, Lombardy, Rezzato (Brescia)	A. Hallgass / 07.2012 / 3 (FGC 42976)	CAN-2	*M. cantiana*	IT-COI 10	2	MG208931	IT-16S 4	3	MG209002	IT-H3 9	1	MG209051				AN	31–35
								MG208932			MG209003	IT-H3 10	1	MG209052	IT-ITS2 8	1	MH137981		
											MG209004								
14.	43°15'58.76"N, 11°28'26.20"E	Italy, Tuscany, Podere Grania (Asciano, Siena)	G. Manganelli & L. Manganelli / 15.10.2000 / (FGC 12960) (Manganelli et al. 2005)	?	*M.* sp.						AY741419								
15.	44°22'09.98"N, 11°15'11.28"E	Italy, Emilia Romagna, along Fiume Setta, upstream its confluence with Fiume Reno (Sasso Marconi, Bologna)	A. Hallgass / 09.2012 / 3 (FGC 42977)	CAN-3	*M.* sp.	IT-COI 11	1	MG208933	IT-16S 6	1	MG209007	IT-H3 2	1	MG209054	IT-ITS2 9	1	MH137982	SH, AN	13, 40–42
						IT-COI 12	1	MG208934	IT-16S 5	2	MG209005	IT-H3 1	1	MG209053					
						IT-COI 13	1	MG208935			MG209006	IT-H3 11	1	MG209040					
16.	46°36'00.9"N, 12°57'59.7"E	Italy, Friuli-Venezia Giulia, Passo di Monte, Croce Carnico	unknown (Duda et al. 2011; Kruckenhauser et al. 2014; Cadahia et al. 2014)	CAN-3	*M.* sp.			HQ204502=			HQ204543=								
								KF596907			KF596863								
17.	48°15'25.50"N, 16°30'46.38"E	Austria, Breitenlee, abandoned railway station	M. Duda / 09.2015 / 3 (FGC 44020)	CAN-3	*M.* sp.	AT-COI 1	2	MG208936	AT-16S 2	2	MG209009	AT-H3 1	3	MG209055	AT-ITS2 1	1	MH137983	SH, AN	14, 43–46
								MG208937			MG209010			MG209057					
						AT-COI 2	1	MG208938	AT-16S 1	1	MG209008			MG209056					
18.	43°46'11.79"N, 07°22'21.50"E	France, Alpes-Maritimes, Vallée de Peillon, Sainte Thecle	A. Hallgass / 24.10.2011/ 5 (FGC 40320)	CAN-4	*M. cemenelea*	FR-COI 1	1	MG208939	FR-16S 1	4	MG209011	FR-H3 1	1	MG209058	FR-ITS2 1	1	MH137984	SH, AN	15, 47–50
											MG209012	FR-H3 2	1	MG209059					
						FR-COI 2	2	MG208940			MG209013	FR-H3 3	1	MG209060					
								MG208941			MG209014								
						FR-COI 3	1	MG208942											
						FR-COI 4	1	MG208943	FR-16S 2	1	MG209015								
19.	44°38'09"N, 04°15'34"E	France, Ardèche, Jaujac	(Dahirel et al. 2015)	CAN-4	*M. cemenelea*			KF986833											
20.	43°18'59.40"N, 11°30'04.20"E	Italy, Tuscany, La Casella (Asciano, Siena)	G. Manganelli / 04.10.2015 / 3 (FGC 44077)	PAR	*M. parumcincta*	IT-COI 20	2	MG208954	IT-16S 11	2	MG209022	IT-H3 12	3	MG209071				SH, AN	16, 51–55
								MG208955			MG209023			MG209066	IT-ITS2 12	2	MH137985		
						IT-COI 21	1	MG208959	IT-16S 12	1	MG209030			MG209062			MH137986		
21.	43°17'15.33"N, 11°25'19.35"E	Italy, Tuscany, along the road to Medane (Asciano, Siena)	G. Manganelli / 08.10.2000 / (FGC 12956) (Manganelli et al. 2005)	PAR	*M. parumcincta*						AY741418								
22.	43°54'18.00"N, 00°49'13.63"E	Italy, Tuscany, Nievole (Montecatini Terme, Pistoia)	A. Hallgass / 20.10.2013 / 2 (FGC 41562)	PAR	*M. parumcincta*	IT-COI 18	1	MG208949	IT-16S 10	1	MG209020	IT-H3 12	2	MG209067	IT-ITS2 11	2	MH137987	AN	
						IT-COI 20	1	MG208952	IT-16S 11	1	MG209024			MG209063			MH137988		
23.	43°30'19.55"N, 11°38'54.92"E	Italy, Tuscany, Autostrada A1: rest area near Ponte Romita (Pergine Valdarno, Arezzo)	A. Hallgass / 10.2013 / 6 (FGC 41561)	PAR	*M. parumcincta*	IT-COI 19	2	MG208950	IT-16S 12	2	MG209028	IT-H3 12	3	MG20906809068	IT-ITS2 11	2	MH137989	SH	
								MG208951			MG209029			MG209069					
						IT-COI 20	1	MG208953	IT-16S 11	1	MG209021			MG209065					
						IT-COI 21	3	MG208956	IT-16S 12	3	MG209025	IT-H3 13	1	MG209070			MH137990		
								MG208957			MG209026				IT-ITS2 13	1	MH137991		
								MG208958			MG209027								
24.	40°13'25.49"N, 15°52'17.07"E	Italy, Basilicata, along the road from Moliterno to Fontana d’Eboli (Moliterno, Potenza)	A. Hallgass / 2012 / 5 (FGC 42962)	PAR	*M. parumcincta*	IT-COI 14	2	MG208944	IT-16S 8	2	MG209017	IT-H3 12	2	MG209061				SH, AN	56–59
								MG208945			MG209018								
						IT-COI 15	1	MG208946	IT-16S 9	1	MG209019			MG209064	IT-ITS2 10	1	MH137992		
						IT-COI 16	1	MG208947	IT-16S 7	1	MG209016								
						IT-COI 17	1	MG208948											
25.	46°42'10"N, 17°14'38"E	Hungary, Kis-Balaton, about 30 m from the Zala Canal on the underside of goldenrod leaves in the scrub-field	J.R. Pieńkowska / 31.07.2011 / 8 (DCBC) (Pieńkowska et al. 2015)		*M. cartusiana*			KM247376			KM247391	HU-H3 1	1	MG209072	HU-ITS2 1	1	MH137993		
26.	45°46'38"N, 10°30'12"E	Italy: Brescia, Anfo towards Ponte Caffaro, calcareous rocks at branch towards Tre Casali	B. Hausdorf / 19.08.2009 / 1 (ZMH51710-1594) (Pieńkowska et al. 2015, Neiber and Hausdorf 2015)		*M. cartusiana*			KM247389			KM247397								
								KX507189			KX495378								

### DNA extraction, amplification, and sequencing

Small foot tissue fragments of alcohol-preserved snails were used for total DNA extraction with Tissue Genomic DNA extraction Mini Kits (Genoplast) according to the manufacturer’s instructions. The purified total DNA was used as template for amplification by polymerase chain reaction (PCR) of partial sequences of the following genes: mitochondrial cytochrome *c* oxidase subunit I (COI), 16S ribosomal DNA (16SrDNA), nuclear histone H3 (H3) and fragment enclosing partial sequence of 5.8SrDNA and complete sequence of internal transcribed spacer 2 of ribosomal DNA (ITS2). A 5’-end fragment of COI (often called “barcode sequence”) was amplified and sequenced using two degenerate primers F01-R04 (F01 5’-CATTTTCHACTAAYCATAARGATATTGG-3’ and R04 5’-TATAAACYTCDGGATGNCCAAAAAA-3’; Dabert et al. 2010). The 16SrRNA gene was amplified and sequenced using primer pair 5’-CGATTTGAACTCAGATCA-3’ (LR-J-12887, Simon et al. 1994) and 5’-GTGCAAAGGTAGCATAATCA-3’ (Gantenbein et al. 1999). The DNA fragment coding H3 was amplified and sequenced using primer pair H3F-H3R (H3F 5’-ATGGCTCGTACCAAGCAGACVGC-3’ and H3R 5’-ATATCCTTRGGCATRATRGTGAC-3’; Colgan et al. 1998). The fragment enclosing partial sequence of 5.8SrDNA and complete sequence of ITS2 was obtained for analyses using primers NEWS2 (5’-TGTGTCGATGAAGAACGCAG-3’) and ITS2-RIXO (5’-TTCTATGCTTAAATTCAGGGG-3’) (Almeyda-Artigas et al. 2000).

The amplified COI fragments consisted of 650 base pairs (bp). Polymerase chain reactions were performed in a volume of 10 μl according to the modified protocol prepared by the Biodiversity Institute of Ontario for the Consortium for the Barcode of Life (http://barcoding.si.edu/PDF/Protocols_for_High_Volume_DNA_Barcode_Analysis.pdf). Reactions were carried out under the following thermal profile: 1 min at 94 °C followed by 42 cycles of 40 s at 94 °C, 40 s at 53 °C, 1 min at 72 °C, and finally 5 min at 72 °C. The amplified 16SrDNA fragments were of about 385 positions. The amplification reactions were conducted in a volume of 10 μl according to a previously described procedure (Manganelli et al. 2005). The amplified H3 sequences consisted of 429 bp. PCR reactions (10 μl) were performed according to the procedure described by Colgan et al. (1998). The 585 position-long sequences of regions enclosing 89 positions of 3’-end of 5.8SrDNA and 496 positions of complete sequence of ITS2 were amplified according to procedure described by Almeyda-Artigas et al. (2000).

The PCR products were verified by agarose gel electrophoresis (1% agarose). Prior to sequencing, samples were purified with thermosensitive Exonuclease I and FastAP Alkaline Phosphatase (Fermentas, Thermo Scientific). Finally, the amplified products were sequenced in both directions with BigDye Terminator v3.1 on an ABI Prism 3130XL Analyzer (Applied Biosystems, Foster City, CA, USA) according to the manufacturer’s protocols.

### Phylogenetic inference

All individual sequences were deposited in GenBank (Table 1). The following COI sequences from GenBank were used: HQ204502 (Duda et al. 2011), KF596907 (Cadahia et al. 2014), KF986833 (Dahirel et al. 2015), KM247375 (Pieńkowska et al. 2015) and KX507234 (Neiber and Hausdorf 2015) of *M. cantiana*, as well as KM247376, KM247389 (Pieńkowska et al. 2015) and KX507189 (Neiber and Hausdorf 2015) of *M. cartusiana* (as an outgroup). Regarding 16SrDNA, the following sequences from GenBank were used: AY741419 (Manganelli et al. 2005), HQ204543 (Duda et al. 2011), KF596863 (Cadahia et al. 2014), KJ458539 (Razkin et al. 2015), KM247390 (Pieńkowska et al. 2015) and KX495428 (Neiber and Hausdorf 2015) of *M. cantiana*, AY741418 (Manganelli et al. 2005) of *M. parumcincta* and KM247391, KM247397 (Pieńkowska et al. 2015) and KX49537 (Neiber and Hausdorf 2015) of *M. cartusiana* (as an outgroup). In analysis of H3 relationships the sequence KF596955 deposited in GenBank by Cadahia et al. (2014) was used.

Sequences were edited by eye using the program BioEdit, version 7.0.6 (Hall 1999). The alignments were performed using the Clustal W programme (Thompson et al. 1994) implemented in MEGA 7 (Kumar et al. 2016). The COI sequences and H3 sequences were aligned according to the translated amino acid sequences. The ends of all sequences were trimmed. The lengths of the sequences after cutting were 592 bp for COI, 287 positions for 16SrDNA, 315 bp for H3 and 496 positions for ITS2. The sequences were collapsed to haplotypes (COI and 16SrDNA) and to common sequences (H3 and ITS2) using the programme ALTER (Alignment Transformation EnviRonment) (Glez-Peña et al. 2010). Gaps and ambiguous positions were removed from alignments prior to phylogenetic analysis.

Maximum Likelihood (ML) analyses were performed with MEGA 7. For each alignment file best nucleotide substitution models were specified according to the Bayesian Information Criterion (BIC): HKY+I for COI sequences (Hasegawa et al. 1985, Kumar et al. 2016), T92+I for 16SrDNA (Tamura 1992, Kumar et al. 2016), TN93+G+I for H3 (Tamura and Nei 1993, Kumar et al. 2016) and JC+G for ITS2 (Jukes and Cantor 1969, Kumar et al. 2016). In parallel, the sequences of COI and 16SrDNA obtained in the present work together with other sequences obtained from GenBank were analysed by the genetic distance Neighbour-Joining method (Saitou and Nei 1987) implemented in MEGA7 (Kumar et al. 2016) using the Kimura two-parameter model (K2P) for pairwise distance calculations (Kimura 1980). Next, mitochondrial sequences of COI and 16SrDNA, and nuclear sequences of H3 and ITS2 were combined and as two data sets subjected to ML analysis. The combined sequences were of length of 879 positions for COI+16SrDNA pair and of 811 positions for H3+ITS2. The specified best nucleotide substitution models for ML analysis according to the Bayesian Information Criterion (BIC) were: HKY+I (Hasegawa et al. 1985, Kumar et al. 2016) for COI+16SrDNA combined sequences and TN93+G+I (Tamura and Nei 1993, Kumar et al. 2016) for H3+ITS2. Finally, sequences of COI, 16SrDNA and H3 were combined for Bayesian inference. Before doing so, uncertain regions were removed from 16SrDNA alignment with the programme GBlocKs 0.91b (Castresana 2000, Talavera and Castresana 2007) with parameters for relaxed selection of blocks. This procedure shortened alignment of 16SrDNA sequences from 287 to 271 positions. The combined sequences with a total length of 1178 positions (592 COI + 271 16SrDNA + 315 H3) were used to infer group phylogeny by Bayesian analysis conducted with the program MrBayes 3.1.2 (Ronquist and Huelsenbeck 2003). *Monacha cartusiana* was added as an outgroup species in each analysis. Using jModelTest2 (Darriba et al. 2012) according to the Bayesian Information Criterion (BIC), we specified a HKY substitution model for our data set (Hasegawa et al. 1985), assuming a gamma distributed rate variation among sites. Four Monte Carlo Markov chains were run for 1 million generations, sampling every 100 generations (the first 250 000 trees were discarded as ‘burn-in’). This gave us a 50% majority rule consensus tree. In parallel, Maximum Likelihood (ML) analysis was performed with MEGA7 (Kumar et al. 2016) and calculated bootstrap values were mapped on the 50% majority rule consensus Bayesian tree.

The haplotype network was inferred with Network 5.0.0.1 to reflect all relationships between COI and 16SrDNA haplotypes. During the analysis, a median-joining calculation implemented in Network 5.0.0.1 was used (Bandelt et al. 1999).

### Morphological study

**Figures 1–2.**
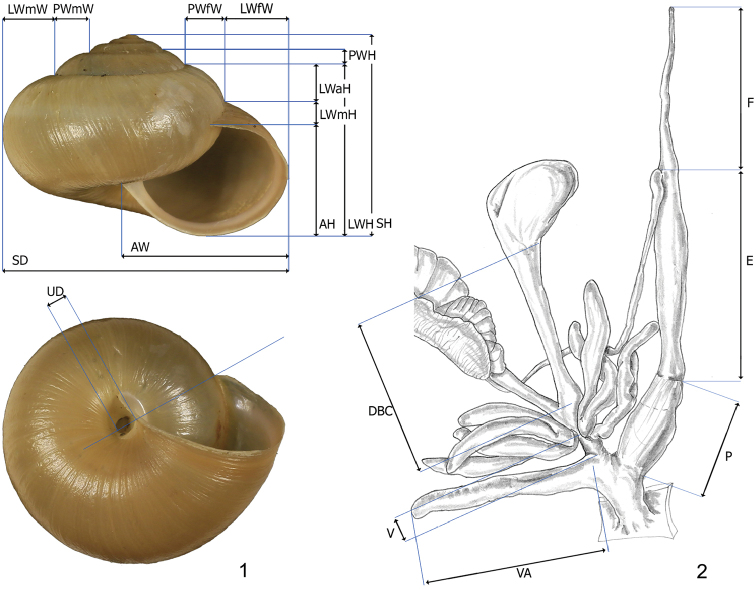
**1** Shell dimensional variables considered for statistical analysis. Abbreviations: AH aperture height, AW aperture width, LWfW last whorl final width, LWmW last whorl medial width, LWH last whorl height, LWaH height of adapical sector of last whorl, LWmH height of medial sector of last whorl, PWH penultimate whorl height, PWfW penultimate whorl final width, PWmW penultimate whorl medial width, SD shell diameter, SH shell height, UD umbilicus diameter **2** Genital dimensional variables considered for statistical analysis. Abbreviations: F flagellum, E epiphallus, P penis, DBC duct of bursa copulatrix, V vagina, VA vaginal appendix.

Approximately 70 specimens of five clades (four lineages of the *M. cantiana* group: CAN-1, CAN-2, CAN-3 and CAN-4; one lineage of *M. parumcincta*) were considered for shell variability (see Table 1). Shell variability was analysed randomly, choosing when possible five adult specimens from each population. Thirteen shell variables were measured to the nearest 0.1 mm using Adobe Photoshop 7.0.1 on digital images of apertural and umbilical standard views taken with a Canon EF 100 mm 1:2.8 L IS USM macro lens mounted on a Canon F6 camera: AH aperture height, AW aperture width, LWfW last whorl final width, LWmW last whorl medial width, LWH last whorl height, LWaH height of adapical sector of last whorl, LWmH height of medial sector of last whorl, PWH penultimate whorl height, PWfW penultimate whorl final width, PWmW penultimate whorl medial width, SD shell diameter, SH shell height, UD umbilicus diameter (Fig. 1).

Approximately 60 specimens of five clades (all lineages of the *M. cantiana* group plus one lineage of *M. parumcincta*) were analysed for anatomical variability (see Table 1). Snail bodies were dissected under the light microscope (Wild M5A or Zeiss SteREO Lumar V12). Anatomical structures were drawn using a Wild camera lucida. Acronyms: BC bursa copulatrix, BW body wall, DBC duct of bursa copulatrix, DG digitiform glands, E epiphallus (from base of flagellum to beginning of penial sheath), F flagellum, FO free oviduct, GA genital atrium, GAR genital atrium retractor, OSD ovispermiduct, P penis, V vagina, VA vaginal appendix (also known as appendicula), VAS vaginal appendix basal sac, VD vas deferens. Six anatomical variables (DBC, E, F, P, V, VA) were measured using a calliper under a light microscope (0.01 mm) (Fig. 2).

Multivariate ordination by Principal Component Analysis (PCA) was performed on shell and genitalia matrices separately in order to determine the degree of correlation between variables and their role in explaining variability. Before PCA, variables were log-transformed to obtain a linear relationship. Since variation in size is the first determinant of biometric variation (e.g., Cadima and Jolliffe 1996, Klingenberg 2016), multivariate morphometrics to distinguish size and shape components by removing isometric effects are nowadays routinely applied in shell biometry studies (Madec et al. 2003, Paquette and Lapointe 2007, Fiorentino et al. 2008, Caruso and Chemello 2009). We therefore performed two PCAs for each data set (shell, genitalia), one on the original matrices and one on the Z-matrices, the latter only consider shape components according to the methods proposed by Cadima and Jolliffe (1996).

Redundancy analysis (RDA; ter Braak 1986) was then applied to the original matrices and Z-matrices in order to detect any multivariate relationships between shell/genitalia variables and the taxonomic assignment. The factors “clade/lineage” were used as constraint factor. An ANOVA-like permutation test for constrained ordination was used to assess the significance (P-value < 0.05) of the constraint for the first two RDA axes. Vegan package (Oksanen et al. 2016) in RStudio 1.0.136 (RStudio Team 2016) was used for processing.

Differences between species for each shell and genitalia characters were assessed through box-plots and descriptive statistics. The significance of differences (P < 0.01) was obtained using analysis of variance (ANOVA); where the test proved significant, an adjusted a posteriori pair-wise comparison between pairs of species was performed using Tukey’s honestly significant difference (HSD) test. All variables were log transformed before analysis.

## Results

### Molecular study

**Figure 3.**
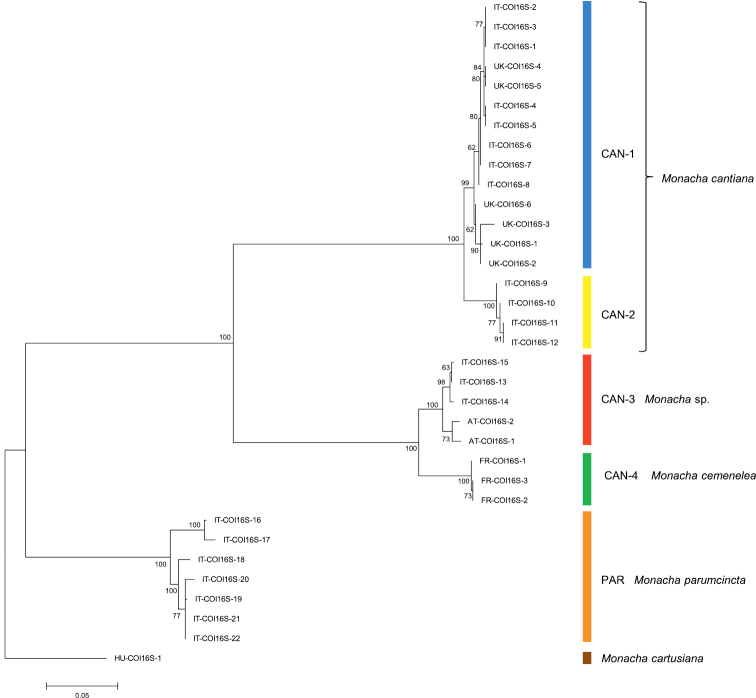
Maximum Likelihood (ML) tree of combined COI and 16SrDNA haplotypes of *Monacha cantiana* group (see: Table 2). Bootstrap support above 50% from maximum likelihood analysis is marked at the nodes. Bootstrap analysis was run with 1000 replicates (Felsenstein 1985). The tree was rooted with *M. cartusiana* combined sequences obtained from GenBank: KM247376 and KM247391.

**Figure 4.**
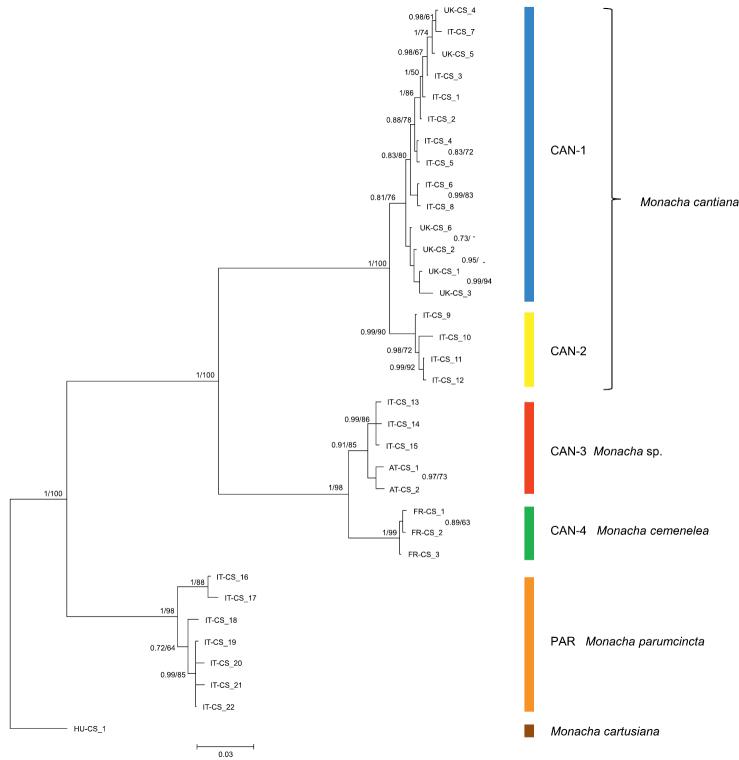
Bayesian 50% majority-rule consensus tree obtained from analysis of the combined data set of COI, 16SrDNA, and H3 sequences (see: Table 2). Posterior probabilities (left) and bootstrap support above 50% from Maximum Likelihood analysis (right) are marked at the nodes. Bootstrap analysis was run with 1000 replicates (Felsenstein 1985). The tree was rooted with *M. cartusiana* combined sequences KM247376, KM247391 and MG209072.

**Figure 5.**
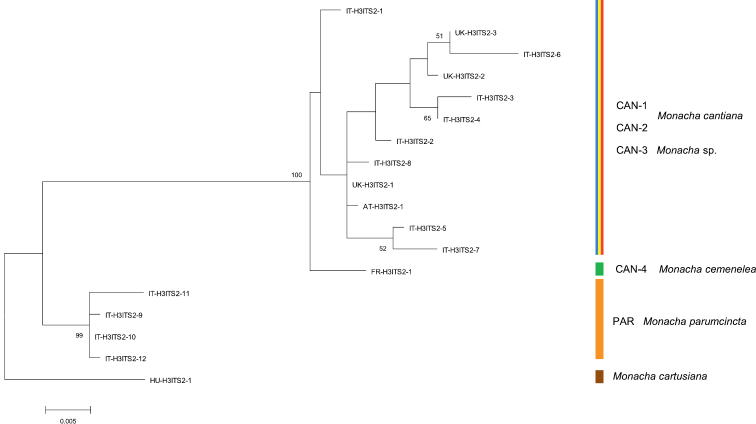
Maximum Likelihood (ML) tree of combined H3 and ITS2 sequences of *Monacha cantiana* group (see: Table 2). Bootstrap support above 50% from maximum likelihood analysis is marked at the nodes. Bootstrap analysis was run with 1000 replicates (Felsenstein 1985). The tree was rooted with *M. cartusiana* combined sequences MG209072 and MH137993.

Thirty-nine and 18 haplotypes of COI and 16SrDNA mitochondrial gene fragments, respectively, as well as 23 and 18 common nucleotide sequences of histone H3 and ITS2 nuclear gene fragments, respectively, were established (Table 1). As a result, 77 sequences of COI as MG208883–MG208959, 71 sequences of 16SrDNA as MG208960–MG209030, 42 sequences of H3 as MG209031–MG209072 and 31 sequences of ITS2 as MH137963–MH137993 were deposited in GenBank (see also Table 1). ML tree for combined sequences of COI and 16SrDNA (Fig. 3, Table 2) as well as Bayesian phylogenetic tree for combined sequences of COI+16SrDNA+H3 gene fragments (Fig. 4, Table 2) clustered the received combined sequences in five clearly separate clades. ML tree of combined sequences of nuclear H3 and ITS2 gene fragments (Fig. 5, Table 2) clustered the combined sequences in three clades.

**Table 2.** Combined Sequences of the following gene sequences: COI+16SrDNA and H3+ITS2 for ML analysis and of COI+16SrDNA+H3 for Bayesian analysis.

Combined Sequence	COI haplotype	16S haplotype	Combined Sequence	H3 sequence	ITS2 sequence	Combined Sequence	COI haplotype	16S haplotype	H3 sequence	Locality (number of specimens)
UK-COI16S-1	UK-COI 12	UK-16S 1				UK-CS_1	UK-COI 12	UK-16S 1	UK-H3 3	UK, Sheffield (1)
UK-COI16S-2	UK-COI 1	UK-16S 1	UK-H3ITS2-1	UK-H3 1	UK-ITS2 2	UK-CS_2	UK-COI 1	UK-16S 1	UK-H3 1	UK, Barrow near Barnsley (3)
UK-COI16S-3	UK-COI 4	UK-16S 1				UK-CS_3	UK-COI 4	UK-16S 1	UK-H3 3	UK, Rotherham (1)
UK-COI16S-4	UK-COI 7	UK-16S 2	UK-H3ITS2-3	UK-H3 5	UK-ITS2 2	UK-CS_4	UK-COI 7	UK-16S 2	UK-H3 5	UK, Sheffield (1)
UK-COI16S-5	UK-COI 7	UK-16S 2	UK-H3ITS2-2	UK-H3 4	UK-ITS2 2	UK-CS_5	UK-COI 7	UK-16S 2	UK-H3 4	UK, Rotherham (1)
UK-COI16S-6	UK-COI 2	UK-16S 1				UK-CS_6	UK-COI 2	UK-16S 1	UK-H3 2	UK, Cambridge (1)
IT-COI16S-1	IT-COI 3	IT-16S 1	IT-H3ITS2-3	IT-H3 7	IT-ITS2 3	IT-CS_1	IT-COI 3	IT-16S 1	IT-H3 7	Italy, Latium, Valle dell’Aniene (1)
IT-COI16S-2	IT-COI 3	IT-16S 1	IT-H3ITS2-2	IT-H3 6	IT-ITS2 2	IT-CS_2	IT-COI 3	IT-16S 1	IT-H3 6	Italy, Latium, Valle dell’Aniene (1)
IT-COI16S-3	IT-COI 3	IT-16S 1	IT-H3ITS2-4	IT-H3 8	IT-ITS2 3	IT-CS_3	IT-COI 3	IT-16S 1	IT-H3 8	Italy, Latium, Valle dell’Aniene (1)
IT-COI16S-4	IT-COI 1	IT-16S 1	IT-H3ITS2-5	IT-H3 1	IT-ITS2 4	IT-CS_4	IT-COI 1	IT-16S 1	IT-H3 1	Italy, Latium, Gole del Velino (1)
IT-COI16S-5	IT-COI 1	IT-16S 1				IT-CS_5	IT-COI 1	IT-16S 1	IT-H3 5	Italy, Latium, Gole del Velino (1)
IT-COI16S-6	IT-COI 7	IT-16S 1	IT-H3ITS2-1	IT-H3 3	IT-ITS2 1	IT-CS_6	IT-COI 7	IT-16S 1	IT-H3 3	Italy, Latium, Valle del Tronto (2)
IT-COI16S-7	IT-COI 7	IT-16S 1	IT-H3ITS2-6	IT-H3 4	IT-ITS2 5	IT-CS_7	IT-COI 7	IT-16S 1	IT-H3 4	Italy, Latium, Valle del Turano (1)
IT-COI16S-8	IT-COI 2	IT-16S 1				IT-CS_8	IT-COI 2	IT-16S 1	IT-H3 3	Italy, Latium, Gole del Velino (1)
IT-COI16S-9	IT-COI 9	IT-16S 3				IT-CS_9	IT-COI 9	IT-16S 3	IT-H3 9	Italy, Venetum, Sorgà (1)
IT-COI16S-10	IT-COI 9	IT-16S 4				IT-CS_10	IT-COI 9	IT-16S 4	IT-H3 4	Italy, Venetum, Sorgà (1)
IT-COI16S-11	IT-COI 10	IT-16S 4				IT-CS_11	IT-COI 10	IT-16S 4	IT-H3 9	Italy, Lombardia, Rezzato (1)
IT-COI16S-12	IT-COI 10	IT-16S 4	IT-H3ITS2-7	IT-H3 10	IT-ITS2 8	IT-CS_12	IT-COI 10	IT-16S 4	IT-H3 10	Italy, Lombardia, Rezzato (1)
IT-COI16S-13	IT-COI 12	IT-16S 5				IT-CS_13	IT-COI 12	IT-16S 5	IT-H3 11	Italy, Emilia Romagna (1)
IT-COI16S-14	IT-COI 11	IT-16S 6	IT-H3ITS2-8	IT-H3 2	IT-ITS2 9	IT-CS_14	IT-COI 11	IT-16S 6	IT-H3 2	Italy, Emilia Romagna (1)
IT-COI16S-15	IT-COI 13	IT-16S 5				IT-CS_15	IT-COI 13	IT-16S 5	IT-H3 1	Italy, Emilia Romagna (1)
IT-COI16S-16	IT-COI 14	IT-16S 8				IT-CS_16	IT-COI 14	IT-16S 8	IT-H3 12	Italy, Basilicata (1)
IT-COI16S-17	IT-COI 15	IT-16S 9				IT-CS_17	IT-COI 15	IT-16S 9	IT-H3 12	Italy, Basilicata (1)
IT-COI16S-18	IT-COI 18	IT-16S 10	IT-H3ITS2-9	IT-H3 12	IT-ITS2 11	IT-CS_18	IT-COI 18	IT-16S 10	IT-H3 12	Italy, Tuscany, Nievole (1)
IT-COI16S-19	IT-COI 19	IT-16S 12				IT-CS_19	IT-COI 19	IT-16S 12	IT-H3 12	Italy, Tuscany, Arezzo (1)
IT-COI16S-20	IT-COI 20	IT-16S 11	IT-H3ITS2-10	IT-H3 12	IT-ITS2 11	IT-CS_20	IT-COI 20	IT-16S 11	IT-H3 12	Italy, Tuscany, Arezzo and Nievole (3)
IT-COI16S-21	IT-COI 21	IT-16S 12	IT-H3ITS2-11	IT-H3 13	IT-ITS2 11	IT-CS_21	IT-COI 21	IT-16S 12	IT-H3 13	Italy, Tuscany, Arezzo (1)
IT-COI16S-22	IT-COI 21	IT-16S 12	IT-H3ITS2-12	IT-H3 12	IT-ITS2 12	IT-CS_22	IT-COI 21	IT-16S 12	IT-H3 12	Italy, Tuscany, Arezzo and La Casella (2)
FR-COI16S-1	FR-COI 1	FR-16S 1	FR-H3ITS2-1	FR-H3 1	FR-ITS2 1	FR-CS_1	FR-COI 1	FR-16S 1	FR-H3 1	France, Alpes-Maritimes, Sainte Thecle (1)
FR-COI16S-2	FR-COI 2	FR-16S 1				FR-CS_2	FR-COI 2	FR-16S 1	FR-H3 2	France, Alpes-Maritimes, Sainte Thecle (1)
FR-COI16S-3	FR-COI 2	FR-16S 1				FR-CS_3	FR-COI 2	FR-16S 1	FR-H3 3	France, Alpes-Maritimes, Sainte Thecle (1)
AT-COI16S-1	AT-COI 1	AT-16S 2	AT-H3ITS2-1	AT-H3 1	AT-ITS2 1	AT-CS_1	AT-COI 1	AT-16S 2	AT-H3 1	Austria, Breitenlee (1)
AT-COI16S-2	AT-COI 2	AT-16S 1				AT-CS_2	AT-COI 2	AT-16S 1	AT-H3 1	Austria, Breitenlee (2)
HU-COI16S-1	KM247376	KM247391	HU-H3ITS2-1	HU-H3 1	HU-ITS2 1	HU-CS_1	KM247376	KM247391	HU-H3 1	Hungary, Kis-Balaton (1)

First clade CAN-1 includes 14 combined sequences in particular trees (Figs 3–4). The clade includes haplotypes and common sequences (Table 1) which have been found in specimens from the following UK populations: Barrow near Barnsley, East Acton, Cambridge, Rotherham and Sheffield, together with those found in specimens from Italian populations from Latium (Gole del Velino, Valle dell’Aniene, Valle del Tronto and Valle del Turano), as well as from Elba island (Tuscan Archipelago). It is noteworthy that sequences of haplotypes UK-COI 1 and UK-16S 1 are identical to sequences KM247375 and KM247390 deposited in GenBank for COI and 16SrDNA of *M. cantiana*, respectively (Pieńkowska et al. 2015). It is also important that UK haplotypes UK-COI 2, UK-16S 2 and UK-ITS 2 are identical to Italian IT-COI 2, IT-16S 1 and IT-ITS2 1, respectively. Moreover, sequences KX507234, KJ458539 and KX495428 deposited in GenBank for *M. cantiana* from Pais Vasco, Sopelana (Neiber and Hausdorf 2015, Razkin et al. 2015), suggest that this Spanish population also belongs to the clade CAN-1. K2P genetic distances between COI and 16SrDNA haplotypes are rather small within the clade CAN-1 (Table 3).

**Table 3.** Ranges of K2P genetic distances for COI and 16SrDNA sequences analysed (mean values in parentheses).

Comparison	COI (%)	16SrDNA (%)
Within *M. cantiana* CAN-1	0.2–2.2 (0.9)	0.7–1.4 (0.7)
Within *M. cantiana* CAN-2	0.3 (0.3)	0.7 (0.7)
Within *M.* sp. CAN-3	0.2–1.9 (1.2)	0.4–2.6 (1.5)
Within *M. cemenelea* CAN-4	0.2–0.5 (0.3)	0.7 (0.7)
Within *M. parumcincta*	0.2–4.6 (2.8)	0.8–4.7 (2.5)
Between *M. cantiana* CAN-1 and *M. cantiana* CAN-2	3.3–5.3 (3.9)	1.8–2.9 (2.5)
Between *M. cantiana* CAN-1 and *M.* sp. CAN-3	17.6–19.3 (18.6)	17.5–18.9 (18.1)
Between *M. cantiana* CAN-1 and *M. cemenelea* CAN-4	17.1–18.9 (18)	20.4–21.9 (21.4)
Between *M. cantiana* CAN-1 and *M. parumcincta*	19.9–22.1 (20.9)	24.7–26.4 (25.5)
Between *M. cantiana* CAN-2 and *M.* sp. CAN-3	17.8–18.2 (18.1)	15.7–17.1 (16.4)
Between *M. cantiana* CAN-2 and *M. cemenelea* CAN-4	18.2–18.7 (18.4)	19.6–20.6 (20.1)
Between *M. cantiana* CAN-2 and *M. parumcincta*	19.7–20.9 (20.3)	23.0–26.5 (24.3)
Between *M.* sp. CAN-3 and *M. cemenelea* CAN-4	5.1–6.2 (5.3)	4.1–5.3 (4.8)
Between *M.* sp. CAN-3 and *M. parumcincta*	17.9–22.0 (19.7)	19.3–21.8 (20.3)
Between *M. cemenelea* CAN-4 and *M. parumcincta*	19.5–21.1 (20.1)	20.4–22.4 (20.8)

Clade CAN-2 (Figs 3–4) includes four COI+16SrDNA combined haplotypes and four COI+16SrDNA+H3 combined sequences. All came from two north Italian populations: Sorgà in Venetum and Rezzato in Lombardy (Table 1). K2P distances between COI and 16SrDNA haplotypes of the clade CAN-2 are very small (Table 3). This CAN-2 clade is not separated from CAN-1 and CAN-3 on the tree of combined nuclear gene sequences (Fig. 5).

Clade CAN-3 is composed of five combined sequences both in COI+16SrDNA (Fig. 3) and COI+16SrDNA+H3 (Fig. 4) trees. It is also not separated in the tree of combined sequences of nuclear H3+ITS2 gene fragments (Fig. 5). The sequences, i.e., COI, 16SrDNA, H3 and ITS2 were from specimens either from Breitenlee in Austria (in Figs 3–5, and Table 1 marked as AT-) or from northern Italy (near Bologna, marked IT-). Sequences deposited in GenBank by Duda et al. (2011), Kruckenhauser et al. (2014) (COI HQ204502, 16SrDNA HQ204543) and by Cadahia et al. (2014) (COI KF596907, 16SrDNA KF596863, H3 KF596955) for *M. cantiana* from the Carnic Alps, Friuli Venezia Giulia, also belong to the CAN-3 lineage. K2P genetic distances of haplotypes within clade CAN-3 varied in a small range (Table 3).

Clade CAN-4 (Figs 3–5) includes three COI+16SrDNA, one H3+ITS2 and three COI+16SrDNA+H3 combined sequences. All were from specimens of a French population in the Maritime Alps near Nice (Sainte Thecle, Table 1). Again K2P genetic distances in this population were small (Table 3). COI sequence KF986833 deposited in GenBank by Dahirel et al. (2015) for *M. cantiana* from Monts d’Ardèche Natural Regional Park near Jaujac (S France) seems to belong to the same clade.

The fifth clade PAR was composed of sequences from specimens identified as *M. parumcincta*. Eight COI and six 16SrDNA haplotypes, as well as two H3 and four ITS2 common sequences were recognised among specimens from four populations from central and southern Italy (Table 1). K2P genetic distances within this clade were larger than for other clades (up to 4.6% in COI haplotypes, Table 3). The clade PAR was clearly separated from other clades in each tree (Figs 3–5). Combined haplotypes IT-COI16S-16 – IT-COI16S-17 from Basilicata (S Italy) seem to form a separate subclade against haplotypes IT-COI16S-18 – IT-COI16S-21 from three other populations in Tuscany (Fig. 3).

K2P genetic distances between COI and 16SrDNA haplotypes are summarised in Table 3. The smallest distances are between haplotypes of CAN-1 and CAN-2 clades; however they are larger than distances within these clades. The largest K2P distances between COI sequences separate clade of haplotypes found in *M. parumcincta* from all other clades (by ca. 20–25 %). Very large distances also separate clade CAN-1 from clades CAN-3 and CAN-4 (COI 18.0% and 18.6%, respectively). Distances between clade CAN-2 and CAN-3 on one hand, and CAN-4 on the other, are also large. Only distances between clades CAN-3 and CAN-4 are smaller (COI 5.3%) although they are much larger than within each of these clades.

**Figure 6.**
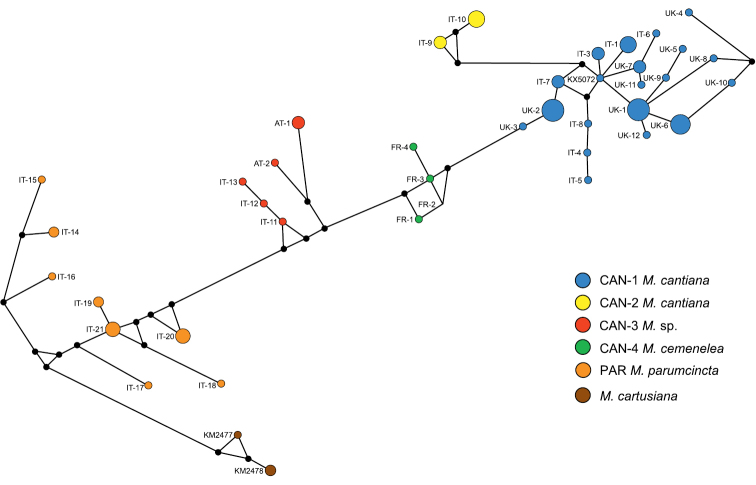
The median-joining haplotype network for COI haplotypes of *Monacha cantiana* group. The colours of the circles indicate *Monacha* species, and their size is proportional to haplotype frequencies. Small black circles are hypothetical missing intermediates.

**Figure 7.**
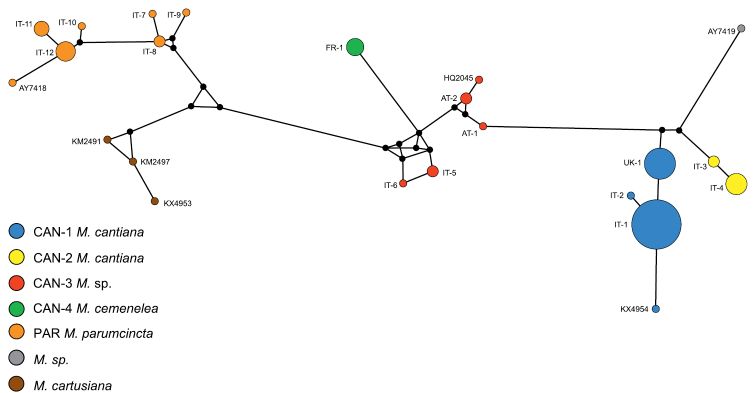
Haplotype network for 16SrDNA of *Monacha cantiana* group. Other explanations as in Figure 6.

Networks of COI (Fig. 6) and 16SrDNA (Fig. 7) confirm separateness of five clades. Clades CAN-1 and CAN-2 are much closer than the others; French haplotypes of clade CAN-4 are separate from the Austrian-Italian CAN-3; clade PAR of *M. parumcincta* haplotypes is differentiated into two subgroups.

### Morphological study: shell

**Figures 8–16.**
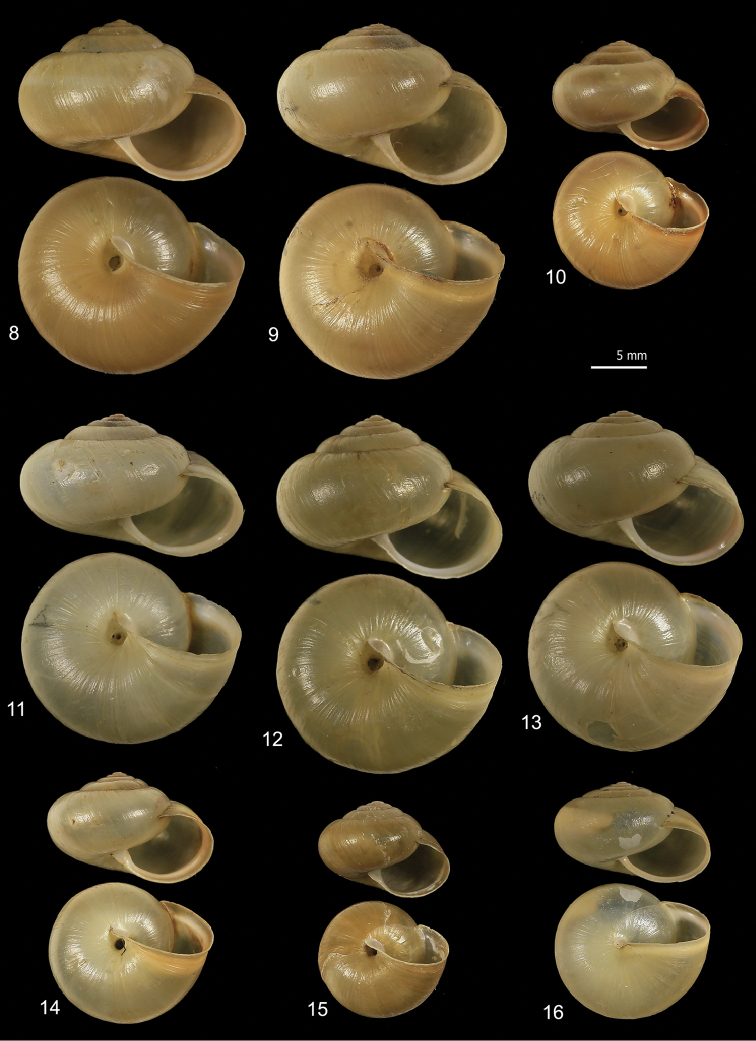
Shell variability in *Monacha cantiana* s.l. group (**8–15**) and *Monacha parumcincta* (**16**). CAN-1 from Valle dell’Aniene (FGC 42973) (**8**), Gole del Velino, near Sigillo (FGC 42960) (**9**), Elba Island, Sant’Ilario in Campo (FGC 23586) (**10**) and Valle del Turano, near Turania (FGC 42969) (**11**); CAN-2 from Sorgà (FGC 42964) (**12**); CAN-3 from Fiume Setta (FGC 42977) (**13**) and Breitenlee (FGC 44020) (**14**); CAN-4 from Vallée de Peillon, Sainte Thecle (FGC 40320) (**15**); PAR from La Casella (FGC 44077) (**16**).

The *M. cantiana* group (clades CAN-1, CAN-2, CAN-3, CAN-4; Figs 8–15) and that of *M. parumcincta* (clade PAR; Fig. 16) have a globose-subglobose shell, variable in colour and size, with roundish aperture and very small or closed umbilicus. The main difference between the two groups consists in the umbilicus (very small, but always open in *M. cantiana* s.l.; closed in *M. parumcincta*). Some populations of *M. parumcincta* have variably evident whitish peripheral and subsutural bands (evident if the last whorl is reddish) and/or a less glossy (more opaque) shell surface.

**Figures 17–18.**
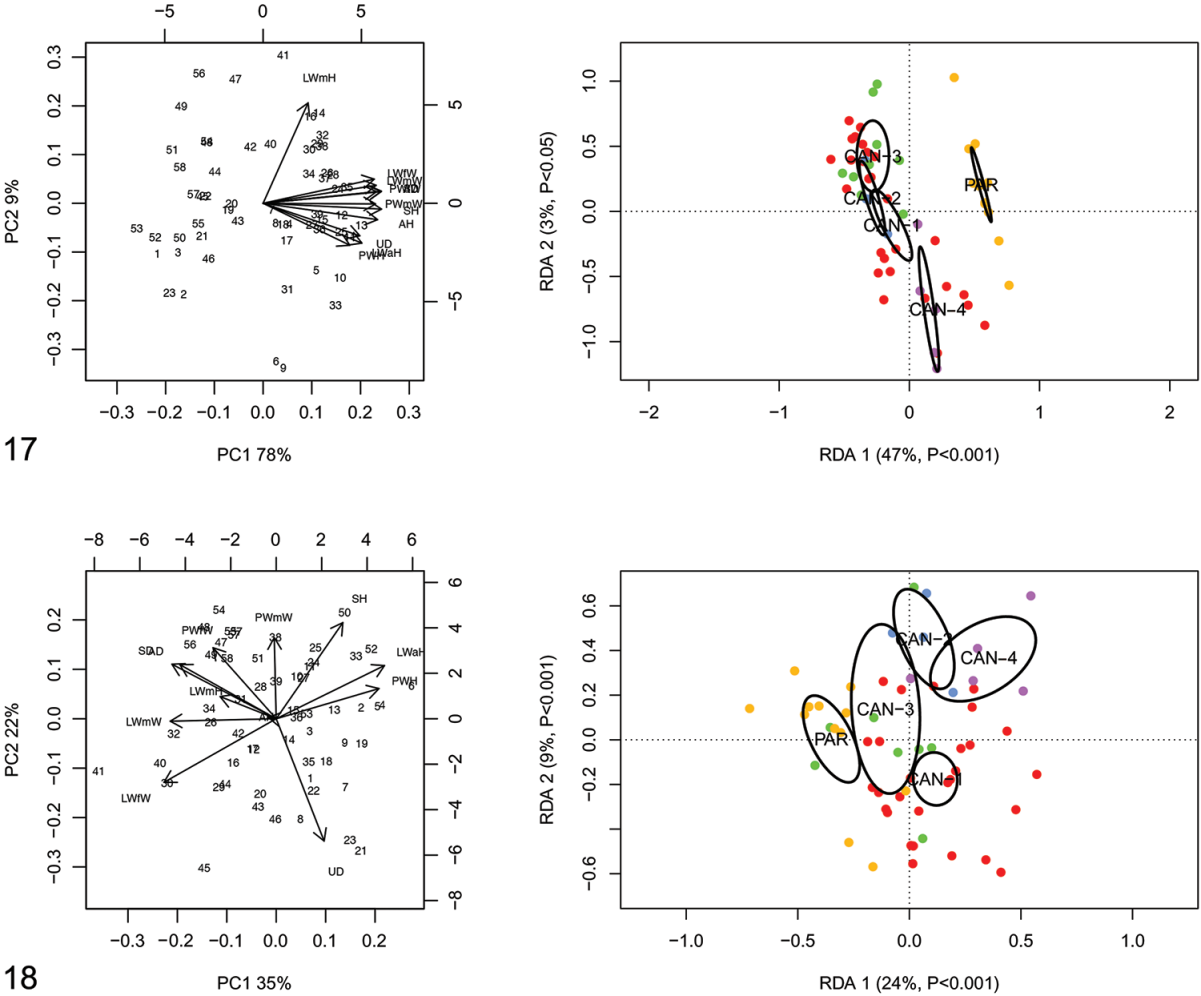
Principal component analysis (PCA) and Redundancy analysis (RDA) with clade constraint applied to the original shell matrix (**17**) and Z-matrix (shape-related)(**18**). Ellipses show the 95% confidence intervals associated with each group.

RDA with “clade/lineage” constraint on the shape and size matrix (Fig. 17) showed that RDA 1 (47%, P < 0.001) separated the groups CAN-1, CAN-2 and CAN-3 from PAR with CAN-4 in intermediate position. The preliminary classic PCA revealed size as the first major source of morphological variation, since PC1 (78%) was a positive combination of all variables. On the contrary, RDA 2 (3%, P < 0.05) showed a statistically significant separation between CAN-4 and the others; no difference was found between the CAN-1, CAN-2 and CAN-3 groups. In this regard, PC2 (9%) accounted for a contrast between LWmH and LWaH / PWH variables. RDA on the shape (Z) matrix (Fig. 18) confirmed a statistically significant separation between PAR and CAN-4 with the large group CAN-1-CAN-2-CAN-3 in intermediate position. Shape-related PCA indicated that LWfW / LWmW / LWmH / SD / AD vs LWaH / PWH were the two principal shape determinants on PC1 and PWmW vs UD on PC2.

**Figure 19.**
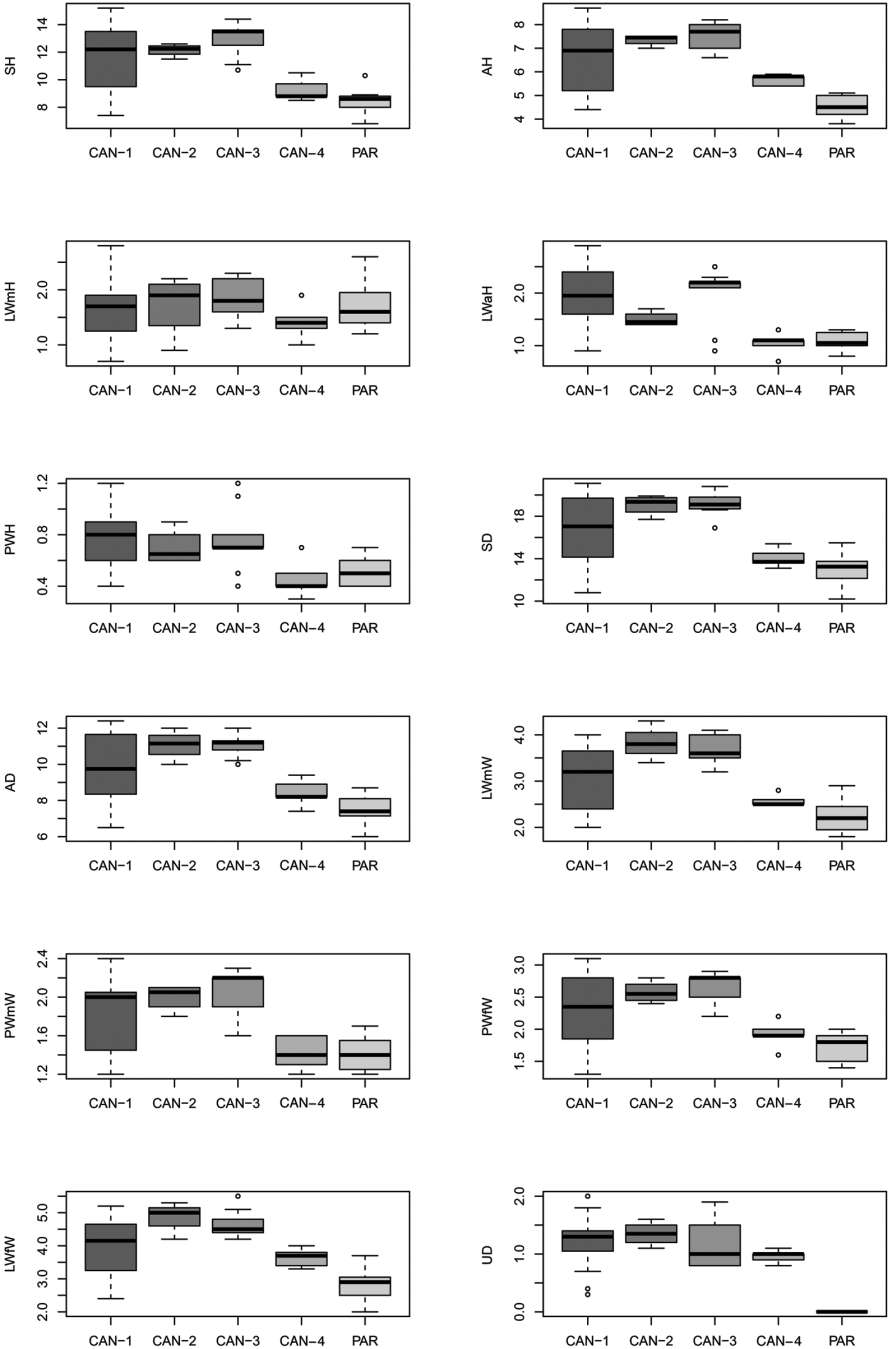
Box plots for shell characters of the five *Monacha* clades investigated. The lower and upper limits of the rectangular boxes indicate the 25^th^ to 75^th^ percentile range, and the horizontal line within the boxes is the median (50^th^ percentile).

Box plots (Fig. 19) prove the poor discriminating value of shell characters in distinguishing species pairs (no character distinguishes more than four clade pairs according to Tukey’s honestly significant difference test). The most recognisable pairs are CAN-1 vs. PAR, CAN-2 vs. PAR, and CAN-3 vs. PAR (11, 9, and 10 significant characters, respectively). Only two significant characters distinguish CAN-1 vs. CAN-4 and only one CAN-3 vs CAN-4 or CAN-4 vs. PAR. No significant character distinguishes CAN-1 vs. CAN-2, CAN-1 vs. CAN-3 or CAN-2 vs. CAN-3 (Table 4).

**Table 4.** Results of Tukey’s honestly significant difference (HSD) test for shell and genitalia characters (in bold Tukey’s post-hoc P < 0.01).

pairs	SH	AH	LWmH	LWaH	PWH	SD
CAN-1 vs CAN-2	0.97573	0.64561	0.99140	0.46817	0.95652	0.47286
CAN-1 vs CAN-3	0.39185	0.18401	0.57940	1.00000	0.99945	0.15274
CAN-1 vs CAN-4	0.05983	0.42921	0.92651	**0.00065**	**0.00567**	0.23583
CAN-1 vs PAR	**0.00001**	**0.00000**	0.97255	**0.00001**	**0.00144**	**0.00030**
CAN-2 vs CAN-3	0.97242	0.99963	0.98207	0.59785	0.98906	1.00000
CAN-2 vs CAN-4	0.11515	0.14765	0.87857	0.38505	0.24954	0.04877
CAN-2 vs PAR	**0.00340**	**0.00008**	1.00000	0.35237	0.39229	**0.00082**
CAN-3 vs CAN-4	**0.00569**	0.02947	0.42967	**0.00414**	0.03203	**0.01007**
CAN-3 vs PAR	**0.00000**	**0.00000**	0.92716	**0.00047**	0.03296	**0.00001**
CAN-4 vs PAR	0.84947	0.12731	0.78714	0.99908	0.96245	0.84026
**pairs**	**AD**	**LWmW**	**PWmW**	**PWfW**	**LWfW**	**UD**
CAN-1 vs CAN-2	0.51068	0.08476	0.82369	0.68103	0.18598	0.87507
CAN-1 vs CAN-3	0.19064	0.03926	0.45194	0.22487	0.12364	0.99947
CAN-1 vs CAN-4	0.33899	0.38635	0.06390	0.44613	0.90473	0.75084
CAN-1 vs PAR	**0.00010**	**0.00008**	**0.00206**	**0.00241**	**0.00002**	**0.00000**
CAN-2 vs CAN-3	1.00000	0.99124	0.99994	0.99975	0.99254	0.86022
CAN-2 vs CAN-4	0.07939	**0.01170**	0.05068	0.16856	0.12920	0.48690
CAN-2 vs PAR	**0.00052**	**0.00002**	**0.01253**	**0.00695**	**0.00003**	**0.00000**
CAN-3 vs CAN-4	0.02106	**0.00660**	**0.00750**	0.03792	0.12320	0.89763
CAN-3 vs PAR	**0.00000**	**0.00000**	**0.00029**	**0.00009**	**0.00000**	**0.00000**
CAN-4 vs PAR	0.60652	0.53369	0.99999	0.86111	0.07669	**0.00000**
**pairs**	**DBC**	**V**	**F**	**E**	**P**	**VA**
CAN-1 vs CAN-2	0.04626	0.99611	0.59664	0.09790	0.14384	**0.00002**
CAN-1 vs CAN-3	0.87421	0.99165	0.91278	0.61442	0.07853	0.03767
CAN-1 vs CAN-4	0.99873	0.47088	0.12512	0.69751	0.65012	0.57764
CAN-1 vs PAR	0.86530	**0.00445**	**0.00938**	**0.00053**	0.95393	**0.00000**
CAN-2 vs CAN-3	0.43904	0.96413	0.97735	0.82401	1.00000	0.14098
CAN-2 vs CAN-4	0.14954	0.46577	0.02416	0.03608	0.04286	0.05841
CAN-2 vs PAR	**0.01497**	0.10864	0.67653	**0.00001**	0.07788	**0.00000**
CAN-3 vs CAN-4	0.89019	0.77914	0.06102	0.21675	0.02722	0.94002
CAN-3 vs PAR	0.48631	**0.01053**	0.24592	**0.00012**	0.04367	**0.00000**
CAN-4 vs PAR	0.99374	**0.00166**	**0.00030**	0.38095	0.93760	**0.00000**

### Morphological study: anatomy

**Figures 20–25.**
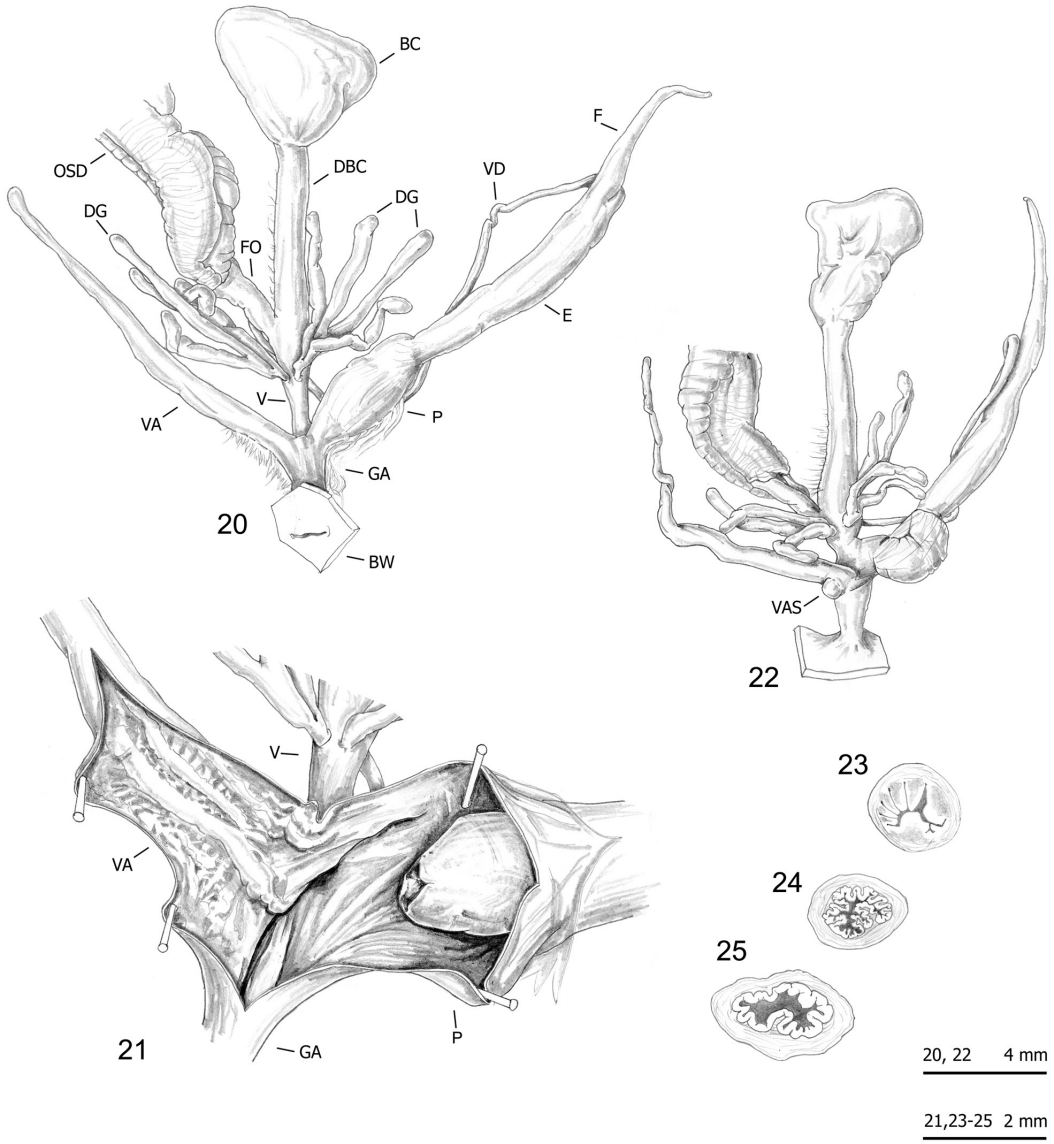
Genitalia (proximal parts excluded) (**20, 22**), internal structure of distal genitalia (**21**) and transverse sections of medial epiphallus (**23**) and penial papilla (**24–25**) of *Monacha cantiana*. CAN-1 from Barrow near Barnsley (FGC 40329) (**20, 22–23, 25**) and East Acton near London (DCBC) (**21, 24**).

**Figures 26–30.**
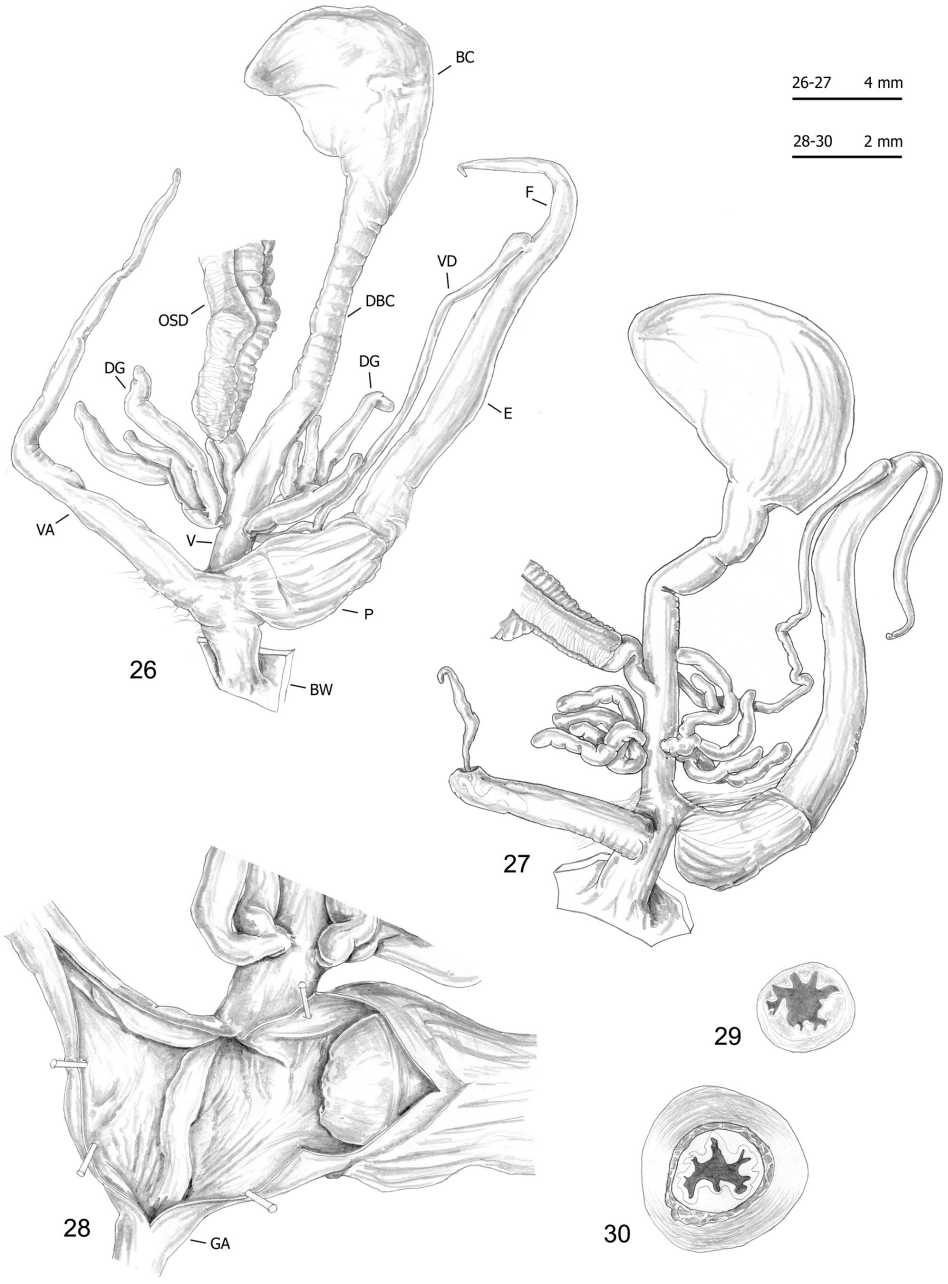
Genitalia (proximal parts excluded) (**26–27**), internal structure of distal genitalia (**28**) and transverse sections of medial epiphallus (**29**) and penial papilla (**30**) of *Monacha cantiana*. CAN-1 from Gole del Velino, near Sigillo (FGC 42960) (**26, 28–30**) and Valle del Turano, near Turania (FGC 42969) (**27**).

**Figures 31–35.**
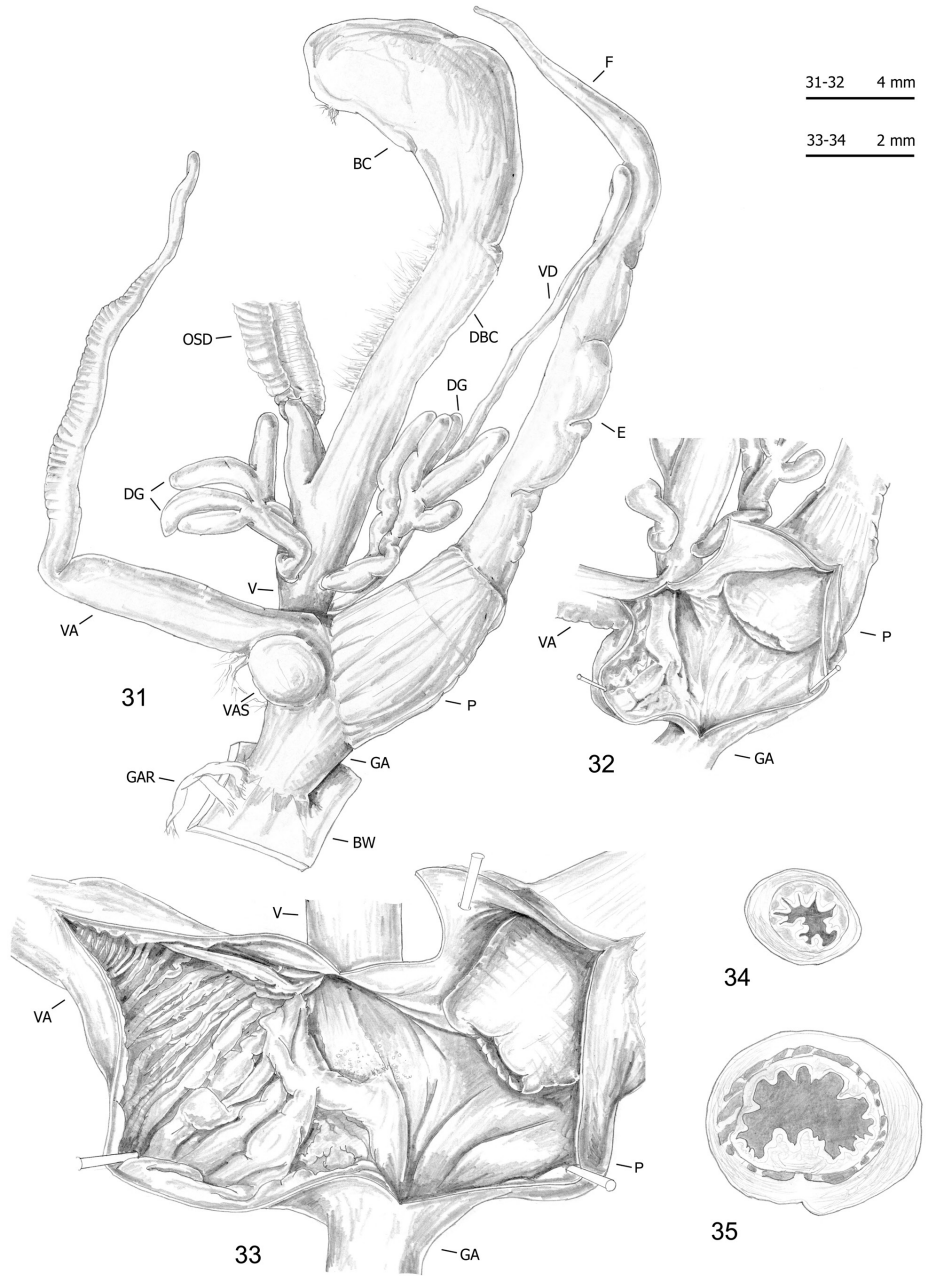
Genitalia (proximal parts excluded) (**31**), internal structure of distal genitalia (**32–32**) and transverse sections of medial epiphallus (**34**) and penial papilla (**35**) of *Monacha cantiana*. CAN-2 from Rezzato (ex. 1: **31–32, 34–35**; ex. 2: **33**) (FGC 42976).

**Figures 36–39.**
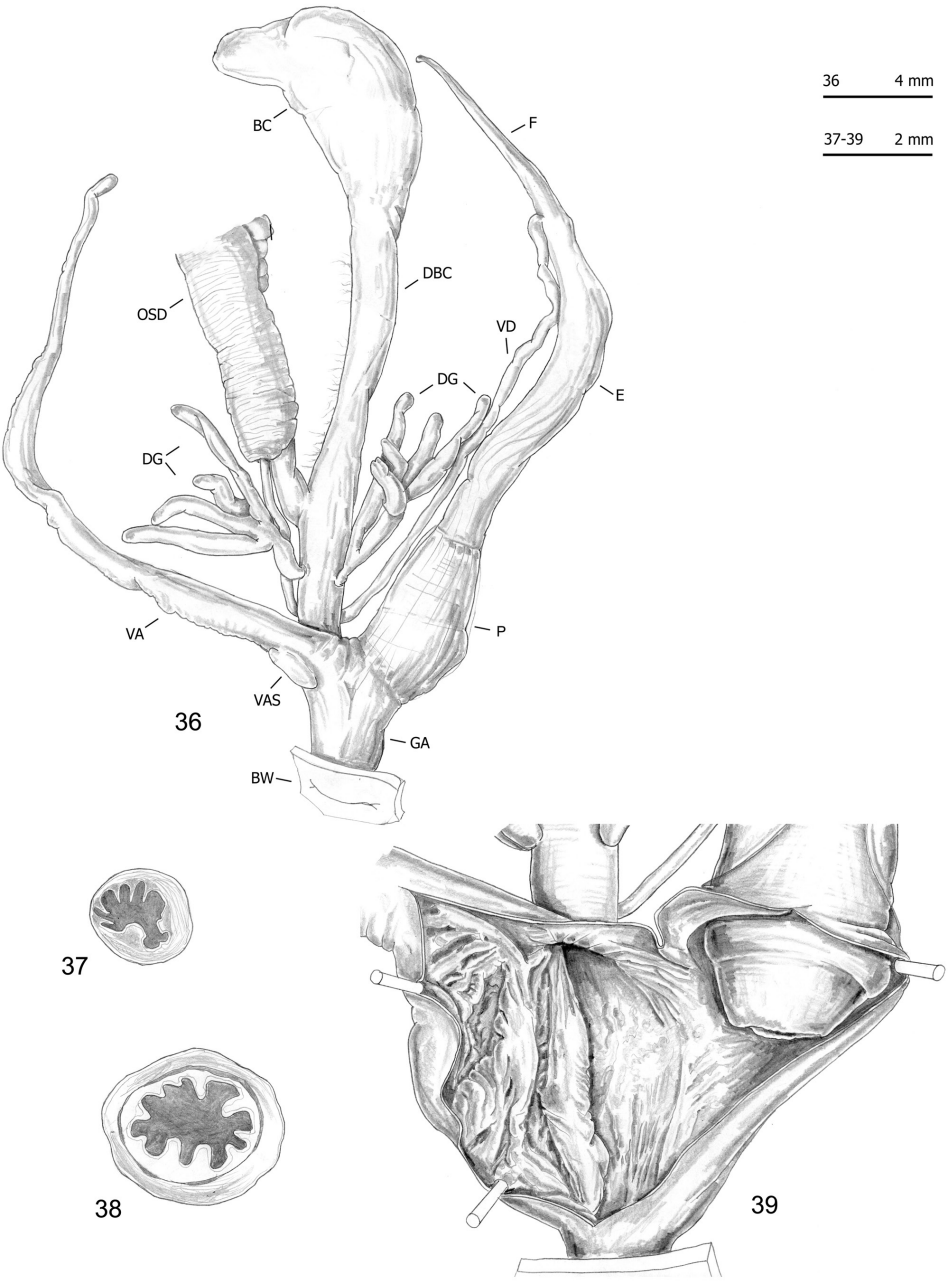
Genitalia (proximal parts excluded) (**36**), transverse sections of medial epiphallus (**37**) and penial papilla (**38**) and internal structure of distal genitalia (**39**) of *Monacha cantiana*. CAN-2 from Sorgà (FGC 42964).

**Figures 40–42.**
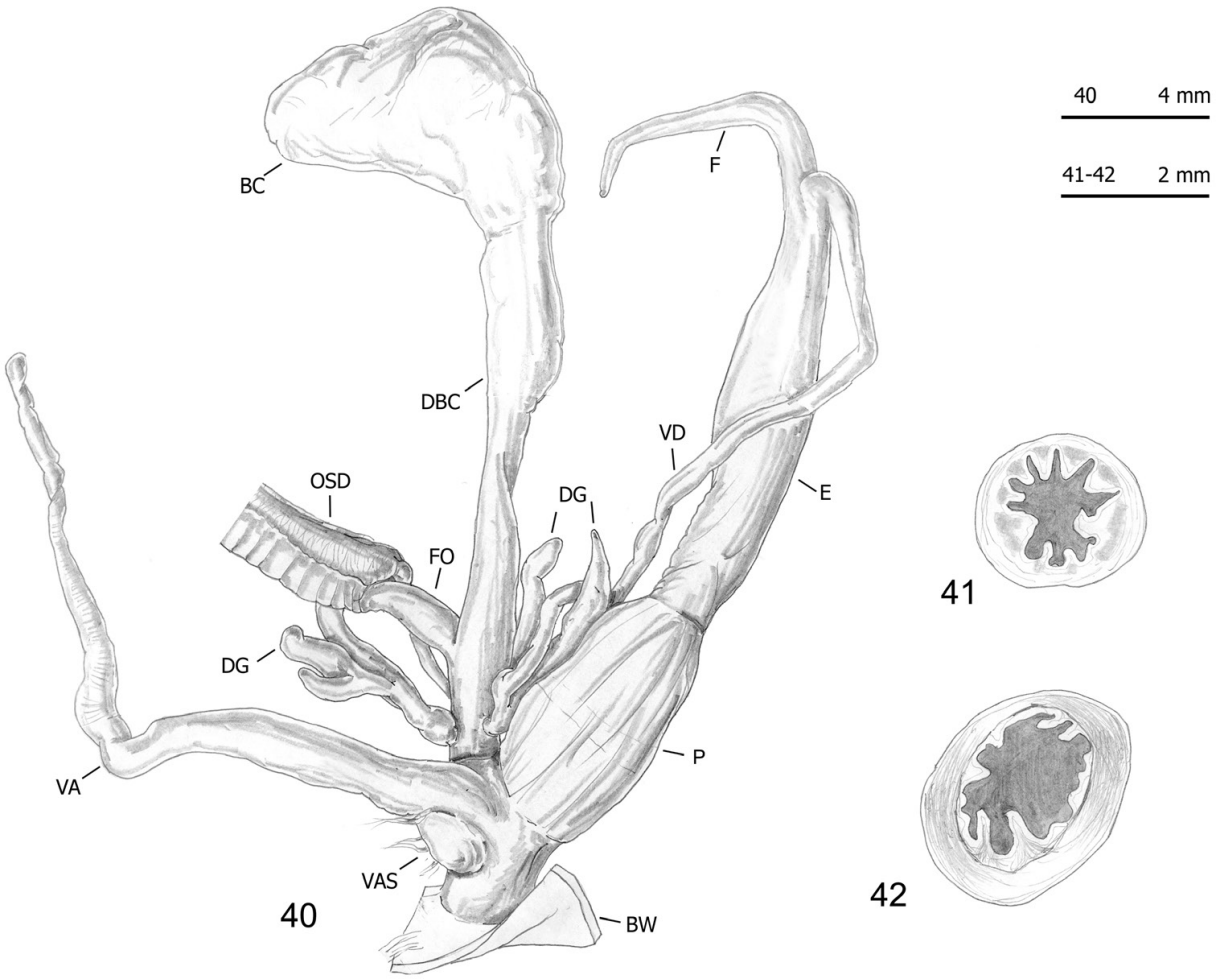
Genitalia (proximal parts excluded) (**40**) and transverse sections of medial epiphallus (**41**) and penial papilla (**42**) of *Monacha cantiana*. CAN-3 from Fiume Setta (FGC 42977).

The bodies (generally pinkish or yellowish white) and mantle (with sparse, variably numerous brown or blackish spots near mantle border or on the lung surface, one larger close to the pneumostomal opening) are very similar in the two species group, whereas the distal genitalia show some diagnostic features (Figs 20–50 vs. Figs 51–59): vaginal appendix or “appendicula” rather long, always with thin walled terminal portion and with variably evident basal sac (i.e., the “sac-like diverticulum of the appendicula vaginalis” first described by Giusti and Manganelli 1987: 135, Fig. 3A, C – in “*M. cantiana*” specimens from Corsica); short, only occasionally with very short terminal portion and always without basal sac in *M. parumcincta*; the vaginal-atrial pilaster (present and variably evident in the *M. cantiana* group; absent in *M. parumcincta*); penial papilla (glans) with central canal wide, thin walled, internally irregularly jagged and with a sort of solid pilaster on one side; central canal connected to external wall of penial papilla by many muscular/connective strings in the *M. cantiana* group; penial papilla with central canal thin walled, internally smooth or slightly jagged, almost completely filled by large invagination; central canal not connected to external wall of penial papilla in *M. parumcincta*.

**Figures 43–46.**
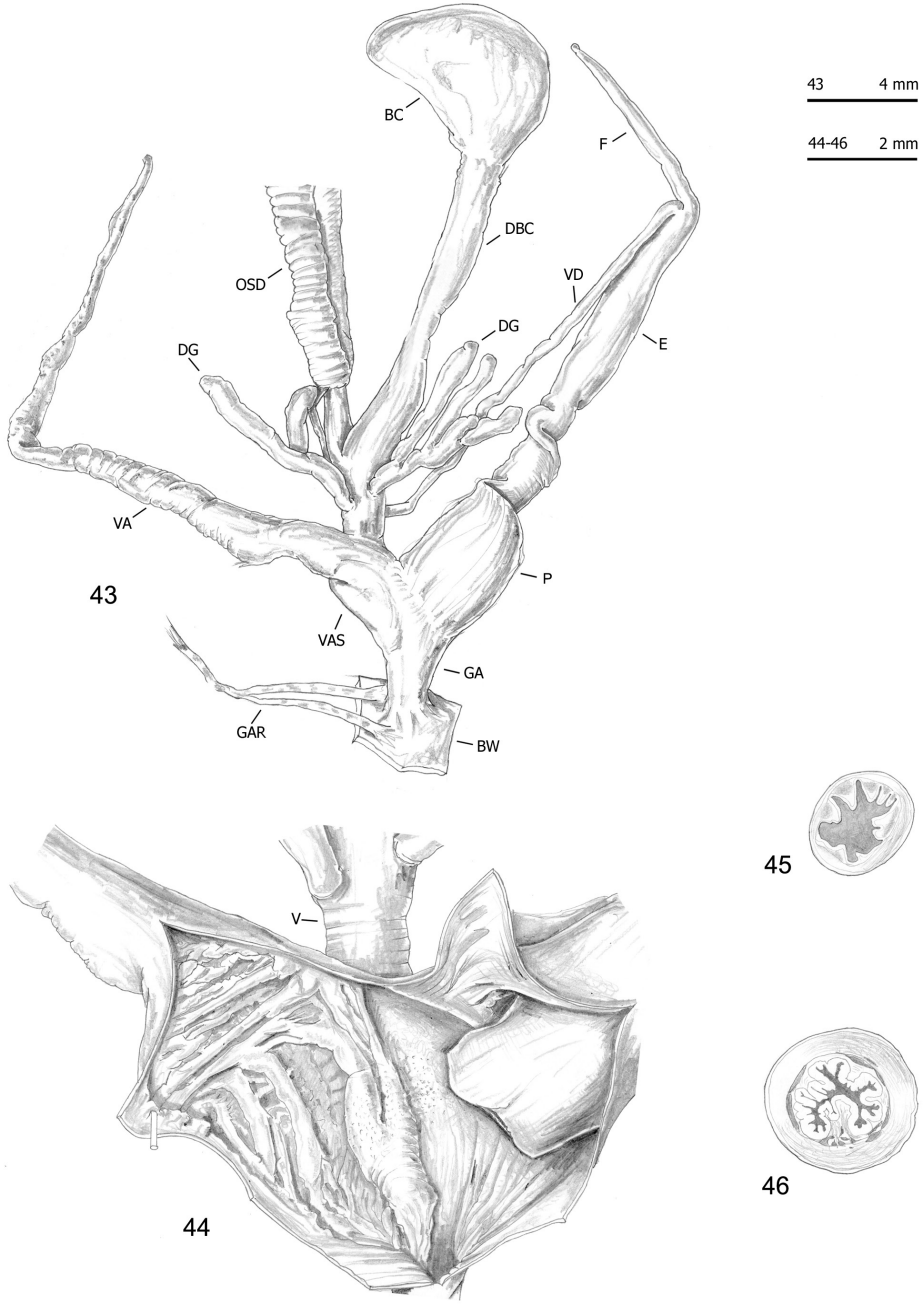
Genitalia (proximal parts excluded) (**43**), internal structure of distal genitalia (**44**) and transverse sections of medial epiphallus (**45**) and penial papilla (**46**) of *Monacha cantiana*. CAN-3 from Breitenlee (FGC 44020).

**Figures 47–50.**
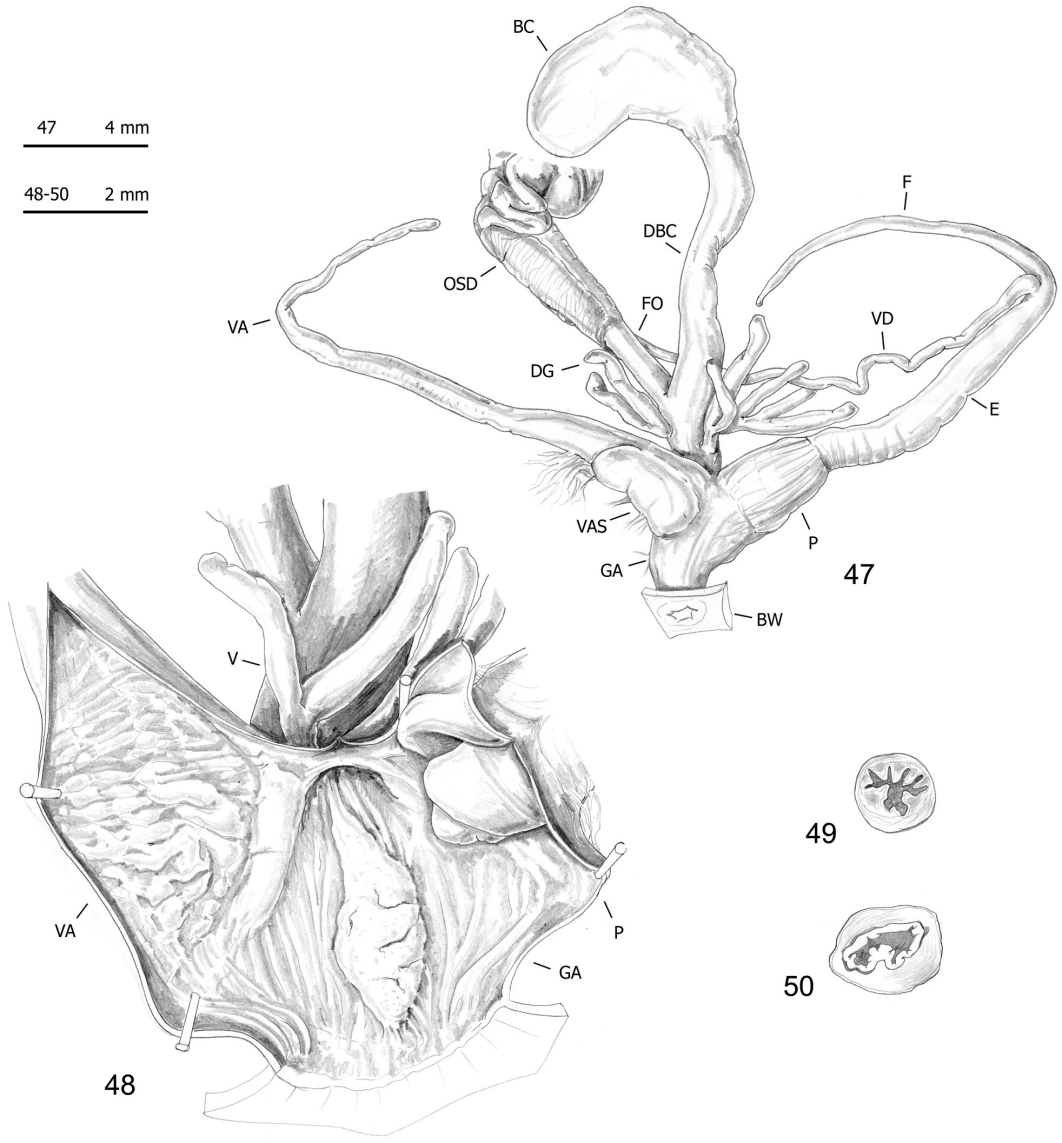
Genitalia (proximal parts excluded) (**47**), internal structure of distal genitalia (**48**) and transverse sections of medial epiphallus (**49**) and penial papilla (**50**) of *Monacha cantiana*. CAN-4 from Vallée de Peillon, Sainte Thecle (FGC 40320).

**Figures 51–59.**
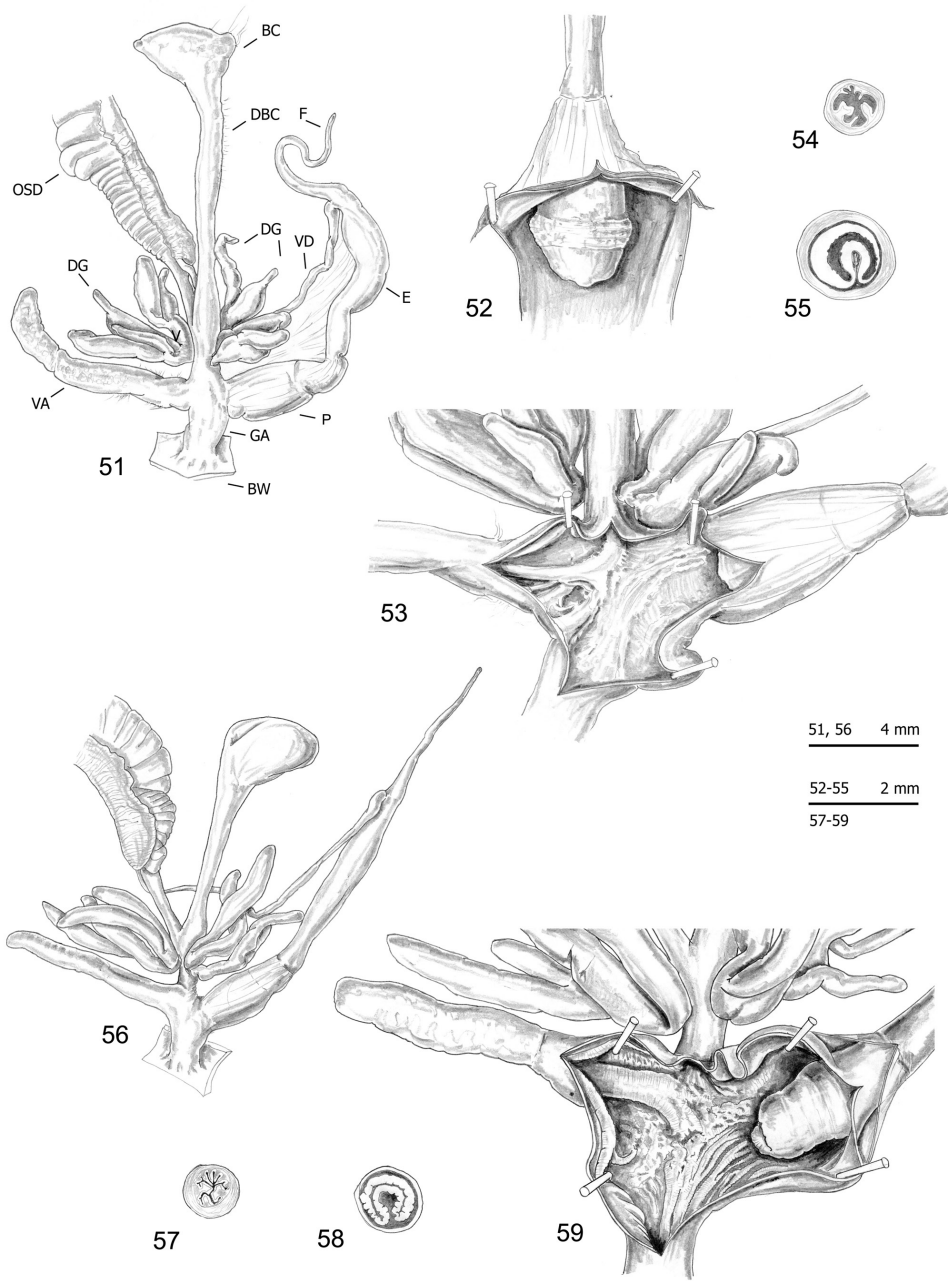
Genitalia (proximal parts excluded) (**51, 56**), internal structure of distal genitalia (**52–53, 59**) and transverse sections of medial epiphallus (**54, 57**) and penial papilla (**55, 58**) of *Monacha parumcincta*. Specimens from La Casella (FGC 44077) (**51–55**) and along the road from Moliterno to Fontana d’Eboli (FGC 42962) (**56–59**).

**Figures 60–61.**
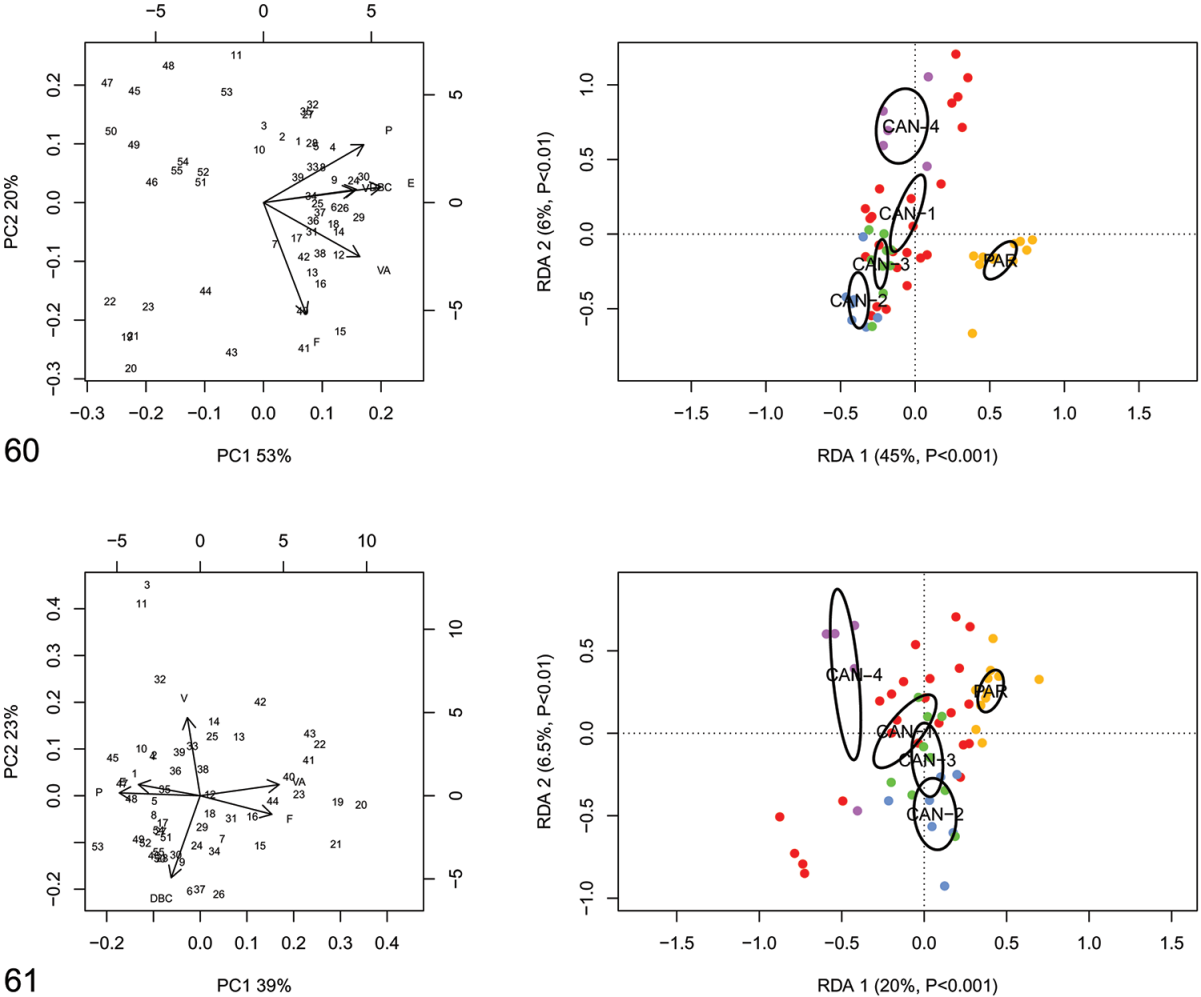
Principal component analysis (PCA) and Redundancy analysis (RDA) with clade applied to the original genitalia matrix (**60**) and Z-matrix (shape-related) (**61**). Ellipses show the 95% confidence intervals associated with each group.

RDA with “clade/lineage” constraint on the shape and size matrix (Fig. 60) showed that RDA 1 (45%, P < 0.001) tended to separate the group CAN-1, CAN-2, CAN-3 and CAN-4 from PAR. The preliminary classic PCA revealed size as the first major source of morphological variation, since PC1 (53%) was a positive combination of all variables. On the contrary, RDA 2 (6%, P < 0.002) showed statistically significant separation of CAN-1, CAN-2, CAN-3 and PAR from CAN-4. In that regard, PC2 (20%) accounted for a contrast between F and P variables. RDA with species constraint on the shape (Z) matrix (Fig. 61) showed that RDA 1 (20%, P < 0.001) confirmed a statistically significant separation between PAR and CAN-4, while the large group CAN-1-CAN-2-CAN-3 remained completely unexplained. Shape-related PCA indicated that VA and F vs E and P were the two principal shape determinants on PC1 and V vs BCD on PC2.

**Figure 62.**
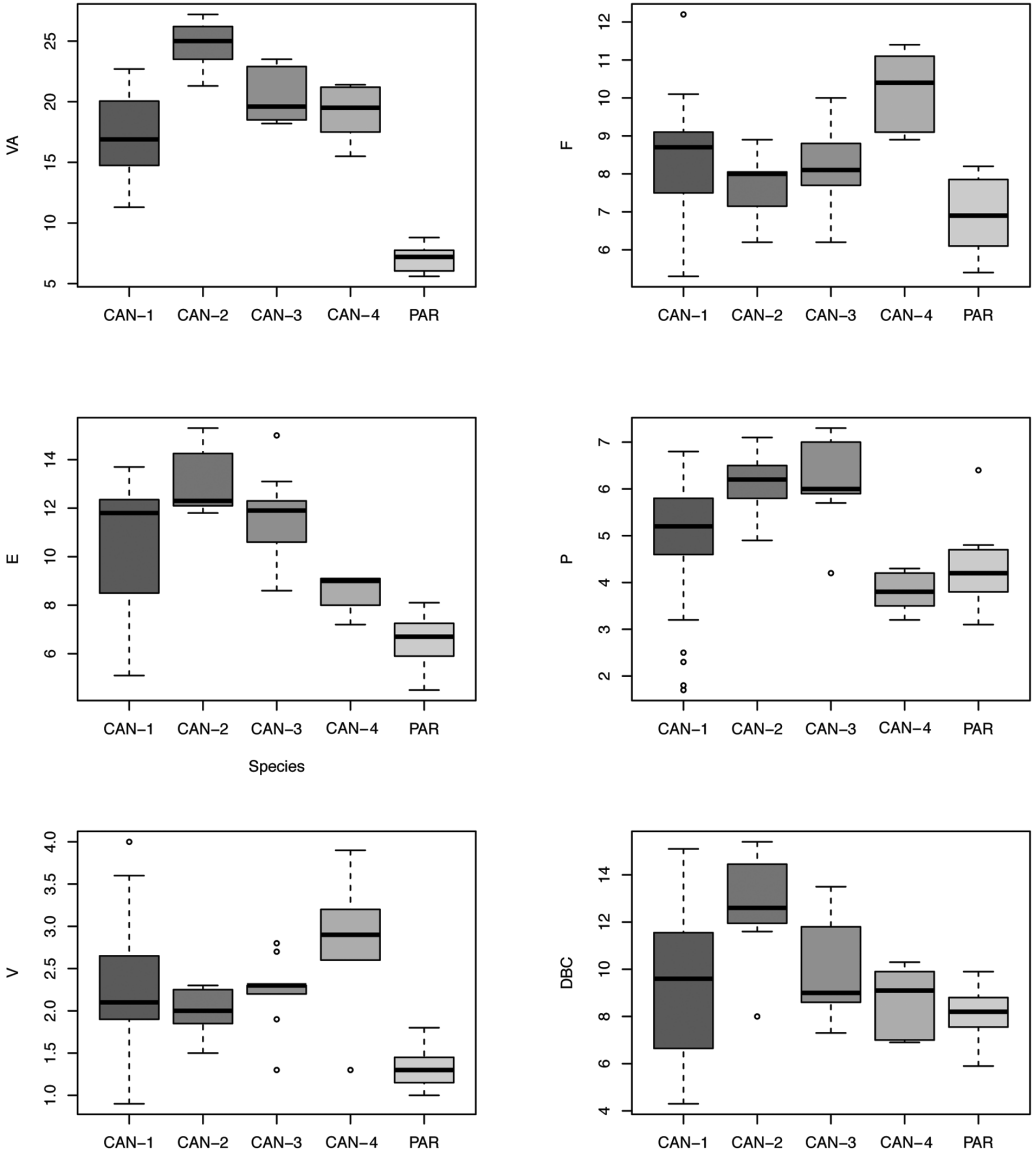
Box plots for anatomical characters of the five *Monacha* clades investigated. The lower and upper limits of the rectangular boxes indicate the 25^th^ to 75^th^ percentile range, and the horizontal line within the boxes is the median (50^th^ percentile).

Box plots (Fig. 62) for anatomical characters showed that VA has the best discriminating value (it distinguishes five clade pairs according to Tukey’s honestly significant difference test), followed by E and V (three pairs). The most recognisable pairs are CAN-1 vs. PAR (four significant characters), CAN-2 vs. PAR, CAN-3 vs. PAR, and CAN-4 vs. PAR (three significant characters). Only one significant character distinguishes CAN-1 vs. CAN-2 and none distinguish CAN-1 vs. CAN-3, CAN-1 vs. CAN-4, CAN-2 vs. CAN-3, CAN-2 vs. CAN-4, or CAN-3 vs. CAN-4 (Table 4).

## Discussion

**Figure 63.**
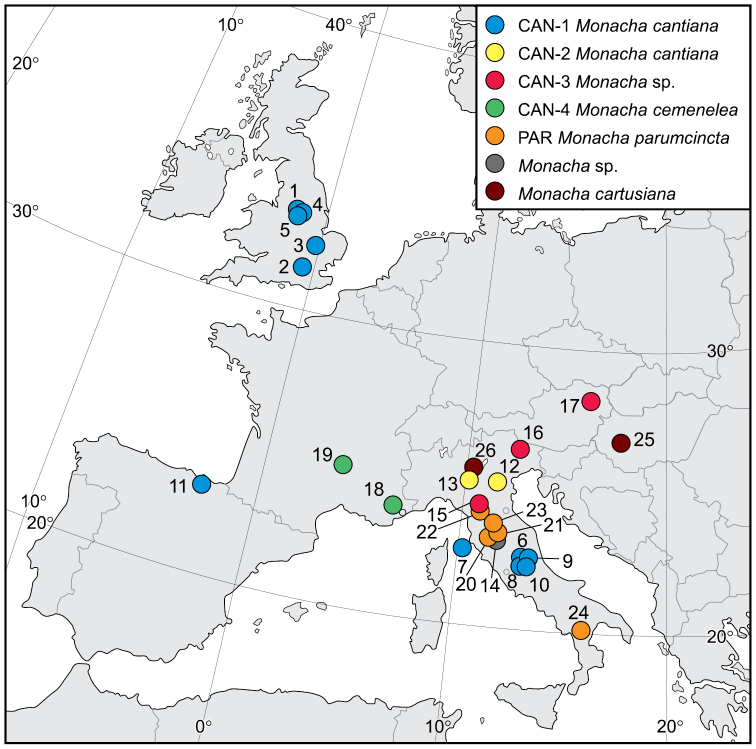
Localities of *Monacha cantiana*, *M. parumcincta* and *M. cartusiana* specimens where they were collected for the research (see Table 1 for locality numbers).

The finding that *M. cantiana*, as usually conceived, actually consists of four distinct lineages (CAN-1, CAN-2, CAN-3, CAN-4) is an absolute novelty. One of these lineages (CAN-1) included most of the populations examined (11 populations). It is widespread and reported from the United Kingdom, Spain and Italy. The other three lineages include only two (CAN-2 and CAN-4) or three (CAN-3) populations, respectively, and at present have a narrow distribution, being known only from two sites in northern Italy (CAN-2), three sites in northern Italy and Austria (CAN-3) and two sites in south-eastern France (CAN-4) (Fig. 63). If these lineages were treated as distinct species, a taxonomical and nomenclatural setting would only be possible for CAN-1 and CAN-4 at present (a definitive framework for the other two requires more research).

Statistical analysis of a series of shell and anatomical characters shows that at least three lineages (CAN-1, CAN-2, CAN-3) cannot be distinguished from each other based on morphology and that one lineage (CAN-4) is only marginally distinct. On the contrary, these four lineages are anatomically well distinct from the *Monacha* species used for comparison (*M. parumcincta*), and three of them (CAN-1, CAN-2, CAN-3) are also conchologically distinct on the basis of many significant characters (11, 9, and 10, respectively). The major bias of morphological analysis was the small sample available for lineages CAN-2, CAN-3, and CAN-4, which prevented a realistic account of their variability.

**Figure 64.**
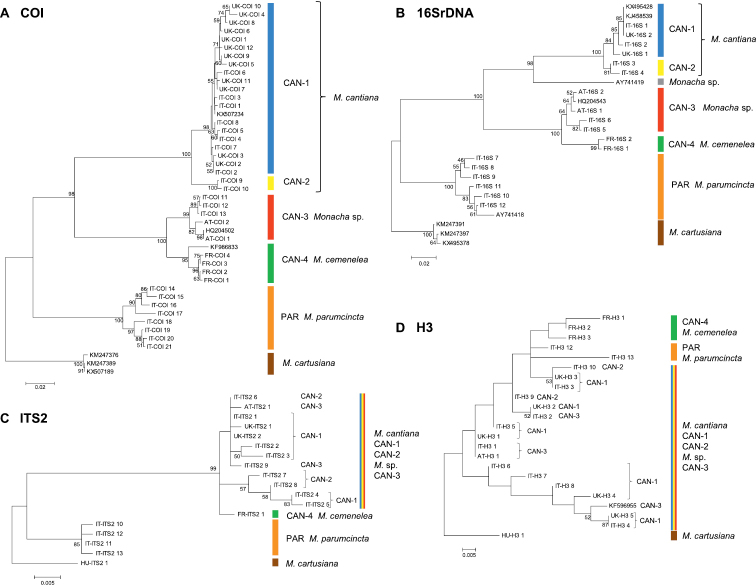
Maximum Likelihood trees of COI, 16SrDNA, H3, and ITS2 sequences of *Monacha cantiana* group. Bootstrap analysis was run with 1000 replicates (Felsenstein 1985). Numbers on branches represent bootstrap support above 50%. **A** the COI sequences of *Monacha cartusiana* KM247389, KM247376 and KX507189 were used as an outgroup, and those of *M. cantiana* KF986833, KX507234 and HQ204502 as reference sequences. 592-bp sequences of new COI haplotypes (Table 1) were shortened to a 556-bp fragment for alignment with the GenBank sequences used as outgroup or references **B** the 16SrDNA sequences of *Monacha cartusiana* KM247391, KM247397 and KX495378 sequences were chosen as outgroup. *M. cantiana* AY741419, HQ204543, KJ458539 and KX495428 as well as *M. parumcincta* AY741418 sequences were used as references. The final dataset contained 287 positions **C** the ITS2 tree was rooted with *Monacha cartusiana* sequence MH137993 **D** the H3 tree was rooted with *Monacha cartusiana* sequence MG209072. *Monacha cantiana* KF596955 was used as a reference.

Sequences characteristic of clade CAN-1 formed a well-separated group in ML and Bayesian trees (Figs 3–5). Although they were all from UK and Italian populations, they are mixed together in the trees without separate branches for UK and Italian populations. Interestingly, three pairs of haplotypes or common sequences are identical: UK-COI 2 / IT-COI 2, UK-16S 2 / IT-16S 1 and UK-ITS2 2 / IT-ITS2 1. This and small K2P genetic distances within this clade (0.9% in COI, 0.5% in 16SrDNA) suggest that the clade represents one taxon. CAN-1 corresponds to the true *M. cantiana* because it is the only clade that includes topotypical English populations. Close relations between the sequences studied (clade CAN-1 in Figs 3–5) support the conclusion that the populations have a common Mediterranean origin (Neiber and Hausdorf 2017), which in view of available fossil record (Kerney et al. 1964, Kerney 1970, Evans 1972), may be postulated to date back to the Roman conquest. The same is also true for the Spanish populations from Pais Vasco (Sopelana), whose sequences (KX507234 and KJ458539 / KX495428), deposited in GenBank for COI and 16SrDNA of *M. cantiana* (Neiber and Hausdorf 2015, Razkin et al. 2015), respectively, were located between our UK and Italian (Latium sites close to Rome) populations in our ML trees (Fig. 64). Nevertheless further studies on molecular characteristics of *M. cantiana* populations from Scotland, N France, N Germany, Belgium, and The Netherlands are necessary in order to test this hypothesis.

The three percent threshold for genetic distance between COI barcode sequences was established by Hebert et al. (2003a, 2003b) as a criterion for the description of a new taxon at species level. There are many papers concerning usefulness of barcoding in taxonomy (e.g., Ebach and Holdrege 2005, Gregory 2005, Goldstein and DeSalle 2010) and showing that 3% threshold should be higher (4% or even higher) for stylommatophoran gastropods (Davison et al. 2009, Sauer and Hausdorf 2012 and references cited therein). Aware of it we think that the slightly exceeded barcode threshold in K2P distances between COI sequences of CAN-1 and CAN-2 clades together with the lack of significant differences in shell (Fig. 19) and genitalia features (Fig. 62), do not permit to introduce a distinct taxon, even at subspecies level. Rather, the K2P distances show that some Italian populations of the *M. cantiana* group are in a process of speciation and differentiation.

The cases of the clades CAN-3 and CAN-4 are completely different, since K2P genetic distances distinguish the haplotypes of these two clades from the others (CAN-1, CAN-2, PAR) and were well above Hebert’s threshold (even enlarged according to Davison et al. 2009). However, due to the lack of differences in anatomical and conchological features between CAN-3 and clades CAN-1 and CAN-2, we treat CAN-3 as mitochondrially distinct lineage only. Any taxonomic conclusion would be premature.

The situation of clade CAN-4 is distinct because this lineage includes a French population which can be considered topotypical of *Theba cemenelea*. Live specimens were collected by one of us (AH) at Sainte Thecle, Vallée de Peillon, a site located 10 km NE of Risso’s original locality: Colline de Cimiez at Nice, now in the urban area of Nice. It was regarded as a junior synonym or at least a subspecies of *M. cantiana* until the early 2000s, when Falkner et al. (2002) separated it again on the basis of the presence of well evident basal sac of the vaginal appendix considered instead absent in *M. cantiana*. Since type material of *T. cemenelea* no longer exists (Chevallier 1976, Arnaud 1977), only designation of a neotype can ensure correct univocal application of Risso’s name. We therefore select a specimen collected at Sainte Thecle in Vallée de Peillon as the neotype. The neotype is deposited in the malacological collection of the Museo di Storia Naturale dell’Accademia dei Fisiocritici, Siena (MOLL/3309). Its shell is illustrated in Fig. 16 and its genital anatomy in Figs 38–41. The separation of CAN-4 (*M. cemenelea*) is strongly supported by nucleotide sequence analysis of both mitochondrial and nuclear genes (Figs 3–5, 64). Therefore haplotypes of COI and 16SrDNA as well as sequences of H3 and ITS2 gene fragments characteristic of specimens from this population have been deposited in GenBank (accession Numbers for FR-COI 1–4: MG208939–MG208943; for FR-16S 1–2: MG209011–MG209015; for FR-H3 1–3: MG209058–MG209060; for FR-ITS2 1: MH137984).

Designation of the neotype is in line with the current concept of this *Monacha* species (e.g., Falkner et al. 2002) i.e., a species distinguished by a well evident basal sac of the vaginal appendix. Contrarily to what has been stated by Falkner et al. (2002), this basal sac is present but smaller or sometimes absent in *M. cantiana*. Moreover this taxonomic setting based on genitalia features is supported by molecular features of mitochondrial and nuclear genes.

A singular sequence AY741419 from Podere Grania, Asciano, Siena deposited in GenBank by Manganelli et al. (2005) for 16SrDNA (Fig. 64B, Table 1) as well as our not yet published molecular results for certain Italian populations (from Alpi Apuane, Tuscany) suggest that Italian *M. cantiana* may include other lineages.

All our results, namely shell (Figs 17–19) and genital (Figs 60–62) structures and molecular evidence of separate clades for each tree (Figs 3–5, 64), show that *M. parumcincta* and *M. cantiana* are distinct taxa. However the definitive taxonomic and nomenclatural setting of *M. parumcincta* is still unclear (see Forcart 1965, Manganelli et al. 1995, Welter-Schultes 2012). This and its infraspecific variation will be the subject of further studies.
